# Effect of condylar chondrocyte exosomes on condylar cartilage osteogenesis in rats under tensile stress

**DOI:** 10.3389/fbioe.2022.1061855

**Published:** 2022-12-06

**Authors:** Yuan Shi, Jiaqi Shao, Zanzan Zhang, Jianan Zhang, Haiping Lu

**Affiliations:** ^1^ Department of Stomatology, Zhejiang Chinese Medical University, Hangzhou, China; ^2^ Department of Stomatology, The Quzhou Affiliated Hospital of Wenzhou Medical University, Quzhou People’s Hospital, Quzhou, China; ^3^ Department of Stomatology, Ningbo No. 2 Hospital, Ningbo, China; ^4^ Department of Dentistry, Center of Orthodontics, Zhejiang University School of Medicine, Sir Run Run Shaw Hospital, Hangzhou, China

**Keywords:** tensile stress, exosomes, condylar chondrocytes, microRNA, growth

## Abstract

**Background:** Functional orthoses are commonly used to treat skeletal Class II malocclusion, but the specific mechanism through which they do this has been a challenging topic in orthodontics. In the present study, we aimed to explore the effect of tensile stress on the osteogenic differentiation of condylar chondrocytes from an exosomal perspective.

**Methods:** We cultured rat condylar chondrocytes under resting and tensile stress conditions and subsequently extracted cellular exosomes from them. We then screened miRNAs that were differentially expressed between the two exosome extracts by high-throughput sequencing and performed bioinformatics analysis and osteogenesis-related target gene prediction using the TargetScan and miRanda softwares. Exosomes cultured under resting and tensile stress conditions were co-cultured with condylar chondrocytes for 24 h to form the Control-Exo and Force-Exo exosome groups, respectively. Quantitative real time PCR(RT-qPCR) and western blotting were then used to determine the mRNA and protein expression levels of *Runx2* and *Sox9* in condylar chondrocytes.

**Results:** The mRNA and protein expression levels of Runx2 and Sox9 in the Force-Exo group were significantly higher than those in the Control-Exo group (*p* < 0.05). The differential miRNA expression results were consistent with our sequencing results. Bioinformatics analysis and target gene prediction results showed that the main biological processes and molecular functions involved in differential miRNA expression in exosomes under tensile stress were biological processes and protein binding, respectively. Kyoto Gene and Genome Data Bank (KEGG) pathway enrichment analysis showed significant enrichment of differentially expressed miRNAs in the mTOR signaling pathway. The differentially expressed miRNAs were found to target osteogenesis-related genes.

**Conclusion:** These results suggest that stimulation of rat condylar chondrocytes with tensile stress can alter the expression levels of certain miRNAs in their exosomes and promote their osteogenic differentiation. Exosomes under tensile stress culture conditions thus have potential applications in the treatment of Osteoarthritis (OA).

## Introduction

Skeletal Class II malocclusion is one of the most common orthodontic clinical disorders, mostly manifesting as underdevelopment of the mandible. In adolescent patients with this disorder, functional orthodontic appliances or orthoses are mostly used for clinical treatment. The biological basis through which functional orthoses guide mandibular growth and remodeling depends on the condyle being an important regulatory site for mandibular growth and development. Mandibular growth and development at the condyle are based on endochondral osteogenesis. Studies (Zhao et al., 2017) have revealed that condylar cartilage can sense mechanical stimuli and convert them into biochemical signals, which are transmitted to condylar chondrocytes and further, through intracellular signaling chains, to downstream proteins in signaling pathways that regulate the expression of genes involved in condyle growth and development.

With progress in research, it has become clearer that exosomes, which are secreted by cells, play an important role in intercellular signaling. Studies have shown that exosomes can regulate biological activities associated with bone marrow mesenchymal stem cell (BMSC) proliferation and differentiation through exosomal miRNAs and also affect the functions of osteoblasts, chondrocytes, and osteoclasts to mediate osteogenesis and bone resorption (Ekström et al., 2013; Wang et al., 2014; Zhang et al., 2015; Qin et al., 2016). However, mature miRNAs in cells are not randomly integrated into exosomes. When cells are in different states, the results of miRNA screening into exosomes will also change. (Akerman et al., 2019). Changes in the environment of cells, such as the presence of compressive or tensile stress, can have a significant effect on the secretion of exosomes from them (Kusuma et al., 2017). Umeda M et al. (Umeda et al., 2015) first reported the role of microRNA-200a in condylar cartilage formation in 2015; they found that the addition of a microRNA-200a inhibitor enhanced the proliferation of condylar chondrocytes and active cartilage growth response and caused significant thickening of the condylar cartilage layer. In 2017, the same team (Yassin et al., 2017) found that transfection of microRNA-200a into condylar chondrocytes resulted in negative feedback regulation of mandibular condylar chondrogenesis. Cao et al. found that inhibition of miR-15b promoted the expression of IGF1, IGF1R, and bcl2, which enhanced the proliferation and inhibited the apoptosis of condylar chondrocytes (Cao et al., 2019). A study also showed that treatment of cartilage defects in mice with exosomes led to an almost complete recovery of cartilage and subchondral bone, in contrast to treatment with saline control, which only led to fibrous repair (Zhang et al., 2016). In summary, studying the effect of exosomes on condylar cartilage osteogenesis and miRNA expression changes in mandibular condylar chondrocytes (MCCs) under tensile stress is important for understanding the mechanism of condylar cartilage osteogenesis regulation under tensile stress as well as the mechanism through which functional orthoses treat skeletal class II malformations.

To the best of our knowledge, this is the first study of the effect of condylar chondrocyte exosomes on condylar cartilage osteogenesis under cellular tensile stress. Here, we aimed to co-culture exosomes with rat condylar chondrocytes under resting and tensile stress conditions to explore the effect of tensile stress on condylar cartilage osteogenesis. We also aimed to analyze the expression profile of exosomal miRNAs under cellular tensile stress and compare it with that of exosomal miRNAs under control conditions. Finally, we aimed to identify differentially expressed exosomal miRNAs between the tensile stress and control conditions as well as the target genes of these miRNAs through bioinformatics analyses.

## Materials and methods

### Establishment and grouping of afterburner models

Ten 2-week-old SD rats (male, SPF class, purchased from Shanghai Experimental Animal Center, Chinese Academy of Sciences, Shanghai,China) were dissected under aseptic conditions to isolate the bilateral mandibular condyles. We exposed the cartilage properly, isolated the rat condylar cartilage, cut it into pieces in PBS, and digested these with 5 ml of trypsin (biosharp, Hefei, China) at 37°C for 30 min. This was followed by digestion with 2 mg/ml type II collagenase (biosharp, Hefei, China) for 120 min at 37°C. The final precipitate was collected after centrifugation at 1800 *g* for 5 min. The resulting cells were resuspended in DMEM (Servicebio, Wuhan, China) containing 10% fetal bovine serum (FBS, Sijiqing, Hangzhou, China), 100 U/ml of penicillin, and 100 μg/ml of streptomycin (biosharp, Hefei, China) at 37°C in a humidified atmosphere containing 5% CO2. The cells were trypsinized after reaching 80% wall apposition. The Passage 0 (P0) generation chondrocytes were stained with toluidine blue (Phygene, Fuzhou, China), observed under an inverted microscope (Leica, Wetzlar, Germany), and photographed.

P2-generation rat condylar chondrocytes were inoculated at a density of 1×10^5^/ml on elastic cell spiking plates (flexcell, Burlington, NC, United States), and the cells were then allowed to grow to 80% fusion and synchronized by changing the serum-free culture medium 24 h before spiking. The cells were desynchronized by replacing the exosome-free DMEM medium containing 10% FBS before the start of cell stressing. A cell stressing device (flexcell, Burlington,NC, United States) was used to apply cyclic tensile stress to the experimental group at a 1 Hz frequency and 10% elongation for 24 h (Ming-Jia et al., 2019). The cells in the control group were exposed to all the same conditions except tensile stress.

### Isolation of MCC-exosomes

The collected medium was centrifuged in an ultra-high-speed refrigerated centrifuge (Beckman Coulter, Brea, CA, United States) at 300 ×*g* and 4°C for 10 min; the precipitated live cells were discarded to retain the upper liquid; this liquid was centrifuged at 2000 ×*g* and 4°C for 10 min and the precipitated dead cells were discarded to retain the upper liquid; this liquid was centrifuged at 10,000 ×*g* and 4°C for 30 min and the precipitated tissue fragments were discarded to retain the upper liquid; the exosome precipitate was obtained after centrifugation at 100,000 *g* for 70 min and 4°C, resuspended, and washed in PBS for total protein extraction or addition to cell culture medium.

### Observation of exosome morphology

Force-Exo and Control-Exo exosomes were each placed on an electron microscope copper mesh with 10 µl drops of exosome PBS suspension, left for 5 min at room temperature, and excess liquid was removed by aspiration with filter paper. A drop of pH 10 2% phosphotungstic acid solution with a pH of 6.5–7.0 was applied to the copper mesh, stained for 2 min at room temperature, and then dried. Excess liquid was removed using filter paper, and placed under a light to dry; then, the morphology and size of the two groups of exosomes were observed by transmission electron microscopy (Hitachi, Tokyo, Japan) and the exosomes were photographed.

### Exosome nanoparticle tracking analysis

To detect the particle size and concentration of exosomes, we places the two groups of exosomes on ice. The exosomes were diluted with 1× PBS and directly placed in a nanoparticle tracking analyzer (PARTICLE METRIX, Luxembourg, Germany) for NTA detection. The results were then recorded.

### Identification of surface-specific protein markers of exosomes by nanofluidics

We took 20 μl of exosomes from each of the two groups and added PBS to dilute the suspensions to 90 μl; we then took 30 μl of diluted exosomes and added 20 μl of fluorescently labeled antibodies (IgG, CD9, CD81) (Proteintech, Chicago, Illinois, United States); these solutions were mixed well in the dark and set aside. They were incubated at 37°C for 30 min. Then we added 1 ml of pre-cooled PBS and ultracentrifuged the solutions at 4°C and 110,000 ×*g* for 70 min. We removed the supernatant, added 1 ml of 4°C PBS, mixed well, and then performed ultracentrifugation at 110,000 ×*g* at 4°C for 70 min. We removed the supernatant and resuspended precipitate in 50 μl of pre-chilled 1× PBS. Sample loading assay (Thermo Fisher Scientific, Waltham,MA, United States) was then added to obtain exosome marker protein index results.

### miRNA sequencing in MCC-Exo

MicroRNA sequencing libraries were prepared using TruSeq Small RNA Sample Prep Kits (Illumina, San Diego, United States). The library construction process is as follows: 1) extraction of total exosome RNA; 2) RNA 3′ adapter ligation; 3) RNA 5′ adapter ligation; 4) RT primers were added for reverse transcription of the synthesized RNA; 5) PCR amplification; 6)Gel-purified amplified cDNA library formation; 7)Validation library formation. The constructed library was sequenced using Illumina Hiseq 2,500, and the sequencing read length was 1 × 50 bp, single-end. The acquired mRNA data were analyzed using the ACGT101-miR software.

### Dyeing tracer of exosomes

PKH26 (Yumebo, Shanghai, China) was added to the two groups of exosomes for staining, and exosomes were co-cultured with condylar chondrocytes for 24 h. The uptake of exosomes by chondrocytes was observed under an optical confocal microscope (Leica, Wetzlar, Germany).

### Detection of *Sox9* and *Runx2* mRNA expression by RT-qPCR

The cells were collected immediately after co-culture of exosomes (100 μg/ml) with condylar chondrocytes in both groups for 24 h. Total RNA was extracted from each group of cells using Trizol (Servicebio, Wuhan, China) according to the manufacturer’s instructions, and its concentration and purity were determined. cDNA was synthesized using a reverse transcription kit (Servicebio, Wuhan, China), and RT-qPCR was performed using a real-time fluorescence quantitative PCR instrument (Bio-rad, Hercules, California, United States). Results were calculated using the 2^-△△Ct^ method, and the primer sequences are shown in Table 3.

### Western blot

The two groups of exosomes (100 μg/ml) and condylar chondrocytes were co-cultured for 24 h, and the co-cultured cells were collected immediately. Total proteins were extracted from cells on ice for 15 min with RIPA Lysis Buffer (Servicebio, Wuhan, China) and centrifuged at 12,000 rpm for 15 min. The BCA Protein Assay kit was used to measure protein concentrations (Servicebio, Wuhan, China). Standard western blotting procedures were followed. The main antibodies used are as follows: *Runx2* (Proteintech, Chicago, Illinois, United States, 1:3,000 dilution), *Sox9* (Proteintech, Chicago, Illinois, United States, 1:1,000 dilution), *β-actin* (Proteintech, Chicago, Illinois, United States, 1:1,000 dilution). The protein expression levels of Runx2 and Sox9 were normalized to *β*-actin expression levels.

### Validation of differential miRNA expression changes by RT-qPCR

The two groups of exosomes were co-cultured with condylar chondrocytes for 24 h before the cells were collected. Trizol was used to extract total RNA from each group of cells. RT-qPCR analysis was performed for miR-199a-5p, miR-25-3p, miR-339-5p, and miR-186-5p, and the results were calculated using the 2^−△△Ct^ method. The primer sequences are shown in Table 3.

### Bioinformatics analysis

The miRNAs that were significantly differentially expressed between the two groups of exosomes were identified using a *t*-test with a threshold of *p <* 0.05 to compare the expression levels of miRNAs between the two groups. The study of biological processes, cellular components, and molecular activities was done using Gene Ontology (GO) function enrichment, and the analysis of pathways involving distinct miRNAs was done using the Kyoto Gene and Genome Database (KEGG). Some miRNAs were predicted to have osteogenesis-related target genes according to TargetScan (v5.0) and Miranda (v3.3a), and the outcome was the intersection of the two predictions.

### Statistical analysis

Data analyses were independently repeated three times, and for normally distributed data, Student’s t-test was used. SPSS 23.0 was used for all statistical analyses (SPSS, Chicago, Illinois, United States). The statistical significance level was set at *p* < 0.05.

## Results

### Identification of rat condylar chondrocytes

**FIGURE 1:**
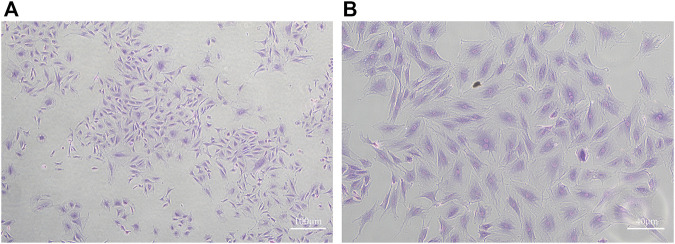
Toluidine blue staining of MCC cells **(A)**: ×40 **(B)** ×100)

Condylar chondrocytes of the P2 generation were identified under an inverted microscope. The cells were large and polygonal and often arranged like paving stones. The nuclei were oval or round (Figures 1A,B).

### Identification of exosomes

**FIGURE 2:**
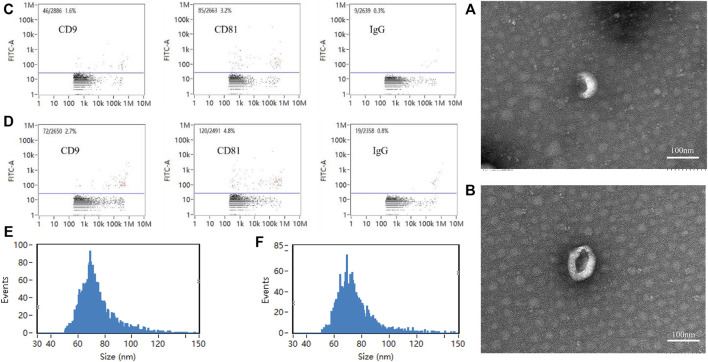
Exosome identification **(A)**: Control-Exo group TEM image. **(B)**: Force-Exo group TEM image. **(C)**: Control-Exo group CD9 and CD81 detection results. **(D)**: Force-Exo group CD9 and CD81 detection results. **(E)**: Control-Exo group exosome particle size distribution. **(F)**: Force-Exo group exosome particle size distribution.

Exosomes of the Force-Exo group and Control-Exo group were collected by ultracentrifugation. The TEM results showed that the MCC-Exos in both groups had a double concave disc-like monolayer membrane structure (Figures 2A,B). The nanoflow results showed that MCC-Exos in both groups expressed CD9 and CD81 membrane protein markers (Figures 2C,D). The NTA test results showed that the particle sizes in both groups were 30–150 nm (Figures 2E,F), which was consistent with exosome characteristics.

### Identification of miRNA transcripts in the Force-Exo group

**FIGURE 3:**
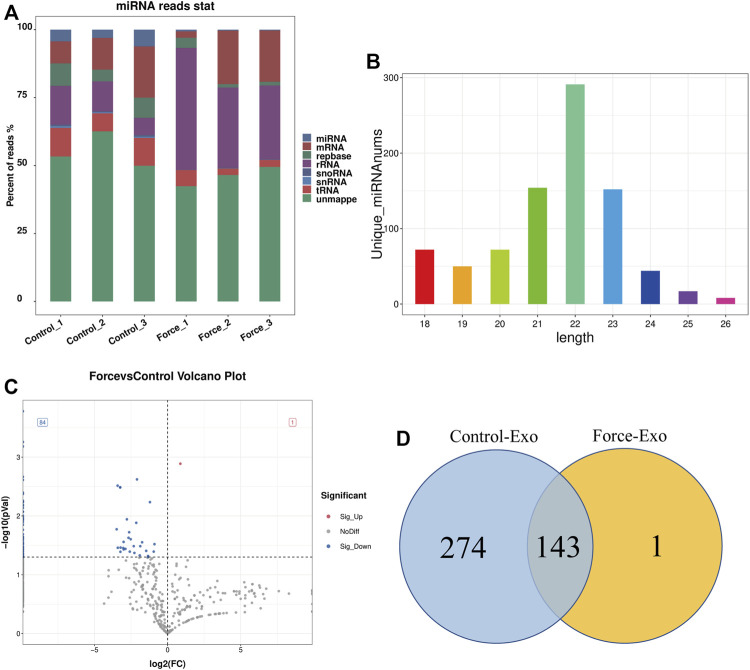
Force-Exo and Control-Exo group miRNA expression profiles. **(A)** Classification of known miRNA genes according to genomic origin. **(B)** Sequence length distribution of all miRNAs **(C)** miRNA expression differences between Force-Exo and Control-Exo groups displayed through a volcano plot. Genes presented as grey dots exhibited non-significantly differential expression between the two MCC-Exo groups, genes presented as red dots exhibited significant up-regulation in the Force-Exo group compared to the Control-Exo group, and genes presented as blue dots exhibited significant down-regulation in the Force-Exo group compared to the Control-Exo group. **(D)** Venn diagram showing the number of miRNAs unique to and common between the Force-Exo and Control-Exo groups.

High-throughput sequencing of miRNAs was performed for three samples each of the Control-Exo and Force-Exo groups. The lengths of the miRNAs sequenced are shown in Figure 3B. Gene statistics are shown in Figure 3A. The miRNA percentages in the Control1, Control2, Control3, Force1, Force2, and Force3 samples were 4.51%, 3.03%, 6.19%, 0.64%, 0.38%, and 0.33%, respectively. A total of 418 miRNAs were detected, of which 144 were expressed in the Force-Exo group, with one being specifically expressed in this group, and 417 were expressed in the Control-Exo group; 143 miRNAs were co-expressed by the two groups (Figure 3D).

### Expression profile of miRNAs in the Force-Exo group

**FIGURE 4:**
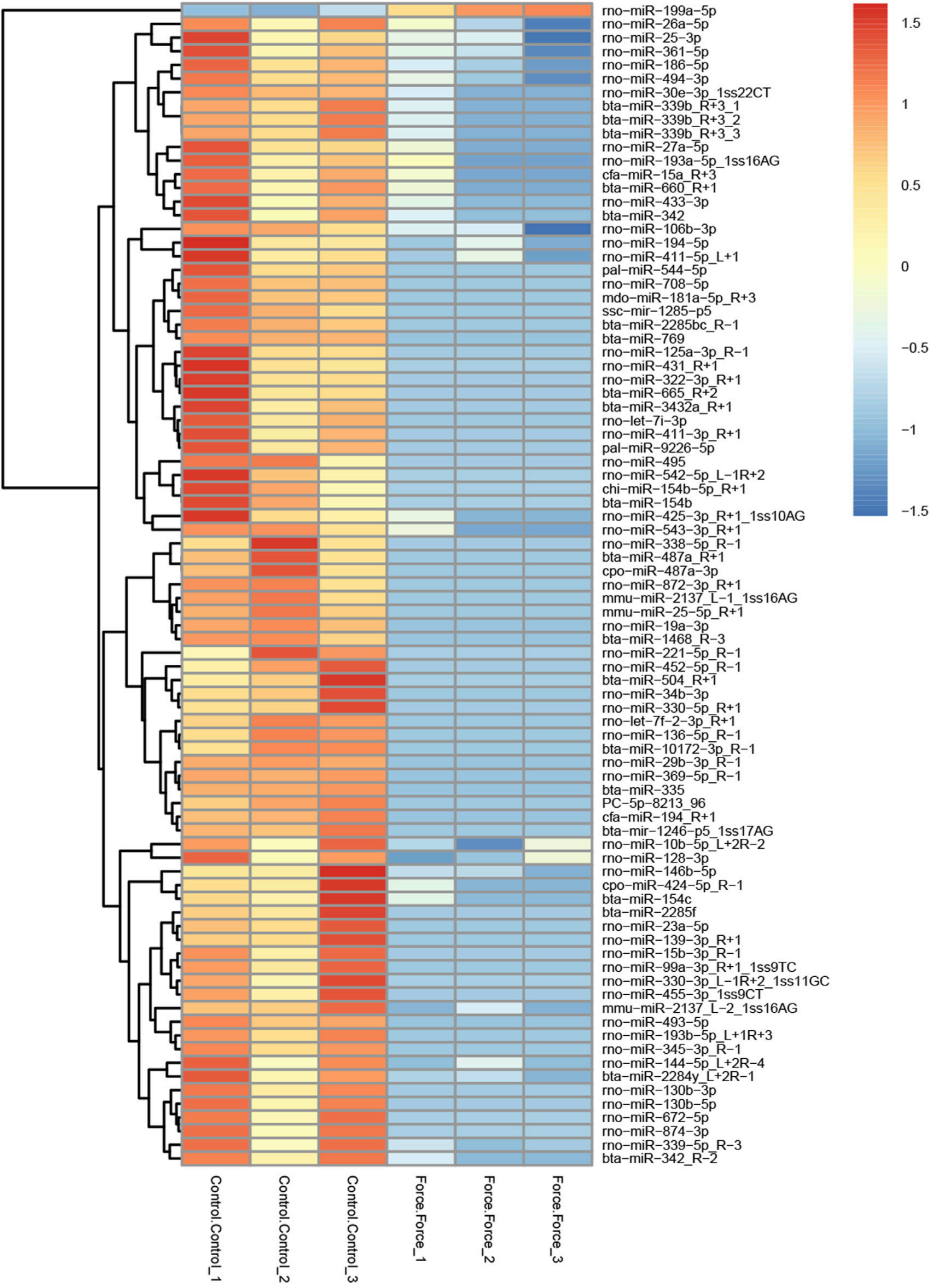
miRNAs showing significantly differential expression levels between the Force-Exo and Control-Exo groups were grouped hierarchically. The sample’s log2 (expression value plus+1) is plotted on the *x*-axis, while gene expression is plotted on the *y*-axis. The color from red to blue indicates the expression of miRNAs from high to low.

A total of 85 miRNAs were significantly differentially expressed (*p* < 0.05) in the Force-Exo group, with one, rno-miR-199a-5p, being upregulated and 84 being down-regulated, compared to the Control-Exo group (Figure 3C). The red dot in the graph indicates the significantly up-regulated miRNA, and the blue dots indicate significantly down-regulated miRNAs. Heat class clustering analysis also showed that miRNA expression levels in the Force-Exo and Control-Exo groups were different (Figure 4). Table 1 lists the four miRNAs that showed a high and significant level of differential expression between the two MCC-Exo groups.

**TABLE 1:** miRNAs with significant differences in expression between the two MCC-Exo groups.

miRNA ID	Control-Exo	Force-Exo	log2(F/C)	*P*	Regulation (Up/Down)
rno-miR-199a-5p	1,334	2,447	1.83	0.0012,943,137	Up
rno-miR-186-5p	519	225	0.43	0.0058,228,208	Down
rno-miR-339-5p	421	167	0.40	0.0498,275,747	Down
rno-miR-25-3p	1,065	422	0.40	0.0479,908,815	Down

The Control-Exo column presents the expression levels of miRNAs, in the Control-Exo group. The Force-Exo column presents the expression levels of miRNAs, in the Force-Exo group.

### Prediction of target genes

Osteogenesis-related target genes of the up-regulated miRNA and several down-regulated miRNAs were predicted. The osteogenesis-related target genes predicted for rno-miR-199a-5p were *Bmp3* and *ECE1*. Among the down-regulated miRNAs, rno-miR-10b-5p was predicted to target samd9, rno-miR-186-5p was predicted to target *Samd8* and *BMP3*, rno-miR-26a-5p was predicted to target *samd1*, and rno-miR-25-3p was predicted to target *BMP3* and *Samd9* (Table 2).

**TABLE 2:** Miranda software-predicted osteogenesis-related target genes of some miRNAs that showed significant differential expression between the two MCC-Exo groups.

miRNA ID	Predicted target genes	miRNA and target gene binding sites	
		Pairing regions between the miRNA and its predicted target gene (underlined)	Pairing percentage (%)
rno-miR-199a-5p	ECE1	3′CGC​CCCCUC​CUCGC​CGUCC​GCG​GA5′	88
		5′CAA​CUUGAG​GAUUC​UAAGG​CGC​CA3′	
	Bmp3	3′CUUGUCCAUCAGAC-UUG​UGA​CCC5′	75
		5′GCCCAGUCAUUAUGAAAC​ACU​GGU3′	
rno-miR-186-5p	Samd8	3′UCG​GGU​UUU​CCUCU​UAA​GAA​AC5′	88
		5′UGU​GCA​UGC​AAAGA​AUU​CUU​UU3′	
	Bmp3	3′UCGGG​UUU-UCCUC-UUA​AGA​AAC5′	81
		5′GCCCC​AAACCGAAGCAAU​UCU​UUG3′	
rno-miR-25-3p	Bmp3	3′AGUCU​G-GCUCUGUUC​ACG​UUAC5′	80
		5′UAAGA​CUUUAUUUAAG​UGC​AAUA3′	
	Smad9	3′AGUCUG-GCUCU​GUUCAC​GUU​AC5′	80
		5′CUAGGCAUGCUG​UAUGUG​CAA​UC3′	
rno-miR-10b-5p	Smad9	3′UGUUUAAGCCAAGAUG​UCC​CAU5′	75
		5′GCAUGUGUUGUUUUAC​AGG​GUA3′	
Rno-miR-339-5p	BMPR2	3′GAG​GAA​CUC​CUG​UCC​CU5′	65
		5′AAU​GGC​AGU​GAC​AGG​GC3′	
	FOSL2	3′GCA​CUC​GAG​GAC​CUC​CUG​UCC​CU5′	87
		5′UCA​GCU​UGG​CCG​GAA​GAC​AGG​GU3′	
rno-miR-26a-5p	Smad1	3′UCG​GAU​AGGACCUA-AUG​AAC​UU5′	87
		5′AUU​GGG​CCUUGCAUGUAC​UUG​AA3′	

### Dye-based tracing of exosomal uptake by rat condylar chondrocytes

**FIGURE 5:**
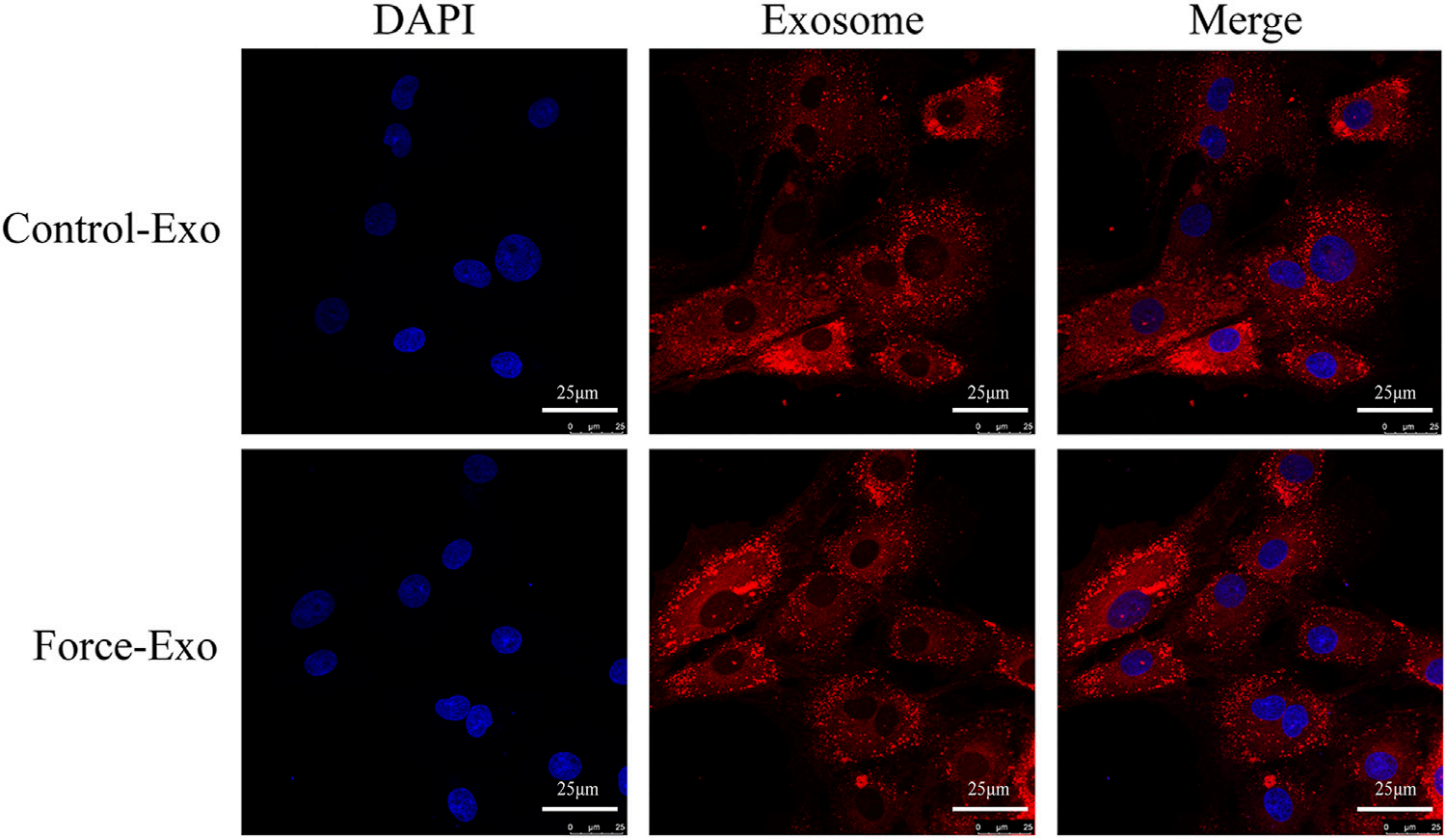
Dye-based tracing of exosomal uptake by rat condylar chondrocytes. DAPI-stained nuclei appear blue, and PKH26-stained exosomes appear red.

To determine whether the exosomes of the Force-Exo and Control-Exo groups could be taken up by rat condylar chondrocytes we stained them with PKH26 and then co-cultured them with rat condylar chondrocytes for 24 h. We then observed these exosomes using laser confocal microscopy and found that condylar chondrocytes had taken up a large number of labeled exosomes (Figure 5).

### Expression of *Runx2* and *Sox9* mRNAs and proteins in condylar chondrocytes co-cultured with the two MCC-Exo groups

**FIGURE 6:**
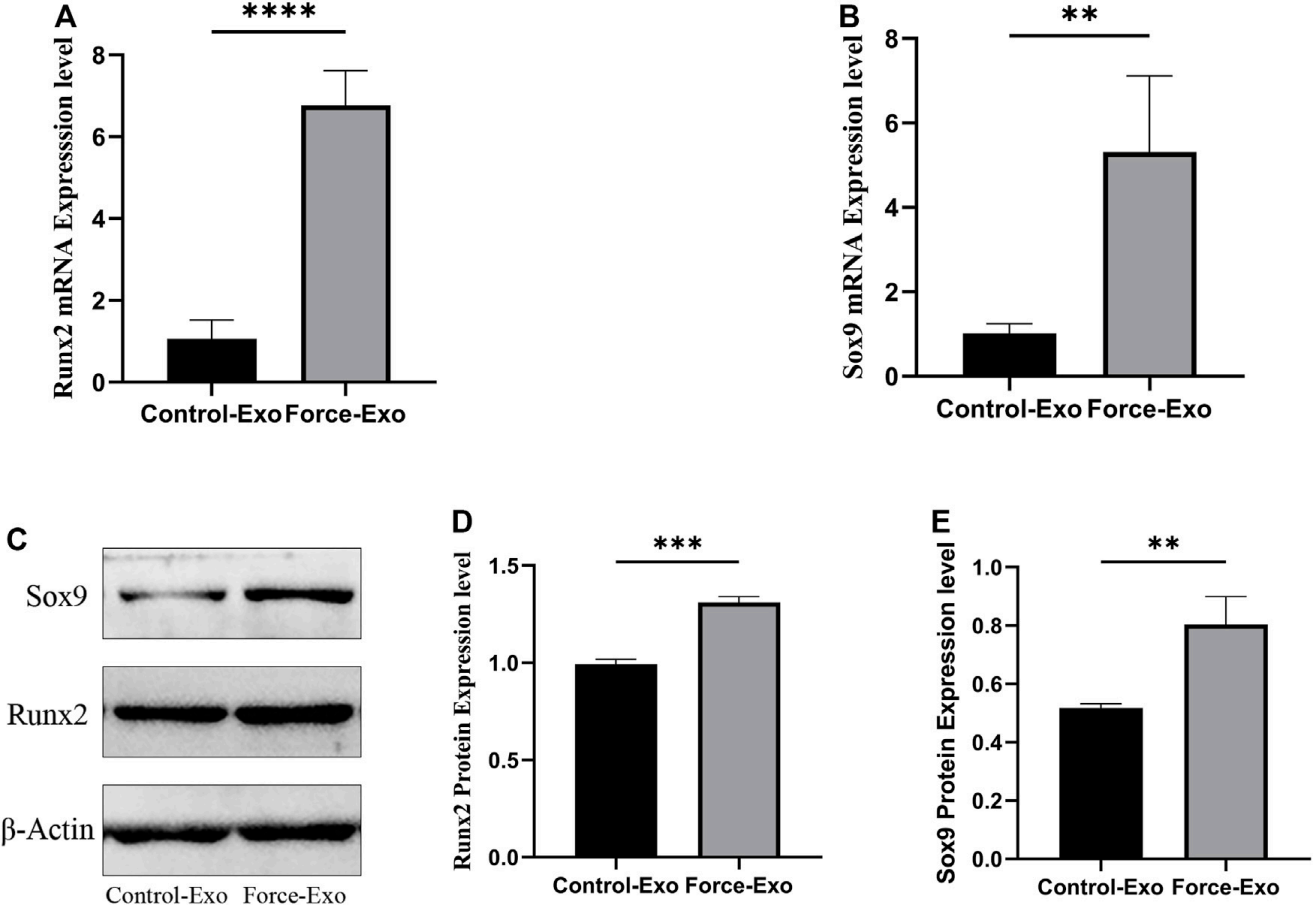
Force-Exo exosomes promoted osteogenic differentiation of condylar chondrocytes. **(A)** The expression level of *Runx2* in condylar chondrocytes of the Force-Exo group was significantly higher than that in condylar chondrocytes of the Control-Exo group, ****p < 0.0001, N = 3 for each group. **(B)** The expression level of *Sox9* in condylar chondrocytes of the Force-Exo group was significantly higher than that in condylar chondrocytes of the Control-Exo group. **p < 0.01, N = 3 for each group. **(C)** The expression levels of *Runx2* and *Sox9* proteins in condylar chondrocytes of the Force-Exo group were significantly higher than those in condylar chondrocytes of the Control-Exo group, **(D,E)** The gray values of the *Runx2* and *Sox9* western blot bands for the Force-Exo group were significantly higher than those for the Control-Exo group, ***p < 0.0005, **p < 0.01, N = 3 for each group.

To verify the effect of Force-Exo exosomes on the osteogenic differentiation of rat condylar chondrocytes, we analyzed the expression levels of *Runx2* and *Sox9* mRNAs and proteins in condylar chondrocytes co-cultured with Force-Exo and Control-Exo exosomes. We found that the mRNA and protein expression levels of *Runx2* and *Sox9* in condylar chondrocytes co-cultured with Force-Exo exosomes were significantly higher than those in condylar chondrocytes co-cultured with Control-Exo exosomes (*p* < 0.05). The sequences of primers employed for mRNA analysis are shown in Table 3 (Figure 6).

**TABLE 3:** miRNA and mRNA primer sequences.

mRNA/miRNA		Primer sequence (5′–3′)
GAPDH	F	CTG​GAG​AAA​CCT​GCC​AAG​TAT​G
	R	GGT​GGA​AGA​ATG​GGA​GTT​GCT
U6	F	CTCGCTTCGGCAGCACA
	R	AAC​GCT​TCA​CGA​ATT​TGC​GT
Runx2	F	CAG​TAT​GAG​AGT​AGG​TGT​CCC​GC
	R	AAG​AGG​GGT​AAG​ACT​GGT​CAT​AGG
Sox9	F	CAA​CTG​AGC​CCG​AGC​CAC​TA
	R	GAG​TTC​TGG​TGG​TCG​GTG​TAG​TC
miR-199a-5p	RT	CTC​AAC​TGG​TGT​CGT​GGA​GTC​GGC​AAT​TCA​GTT​GAG​GAA​CAG​GT
	F	ACA​CTC​CAG​CTG​GGC​CCA​GTG​TTC​AGA​CTA​C
	R	TGGTGTCGTGGAGTCG
miR-186-5p	RT	CTC​AAC​TGG​TGT​CGT​GGA​GTC​GGC​AAT​TCA​GTT​GAG​AGC​CCA​AA
	F	ACA​CTC​CAG​CTG​GGC​AAA​GAA​TTC​TCC​TTT
	R	TGGTGTCGTGGAGTCG
miR-399-5p	RT	CTC​AAC​TGG​TGT​CGT​GGA​GTC​GGC​AAT​TCA​GTT​GAG​CGT​GAG​CT
	F	ACA​CTC​CAG​CTG​GGT​CCC​TGT​CCT​CCA​GGA​G
	R	TGGTGTCGTGGAGTCG
miR-25-3p	RT	CTC​AAC​TGG​TGT​CGT​GGA​GTC​GGC​AAT​TCA​GTT​GAG​TCA​GAC​CG
	F	ACA​CTC​CAG​CTG​GGC​ATT​GCA​CTT​GTC​TCG
	R	TGGTGTCGTGGAGTCG

### Validation of differentially expressed miRNAs by RT-qPCR

**FIGURE 7:**
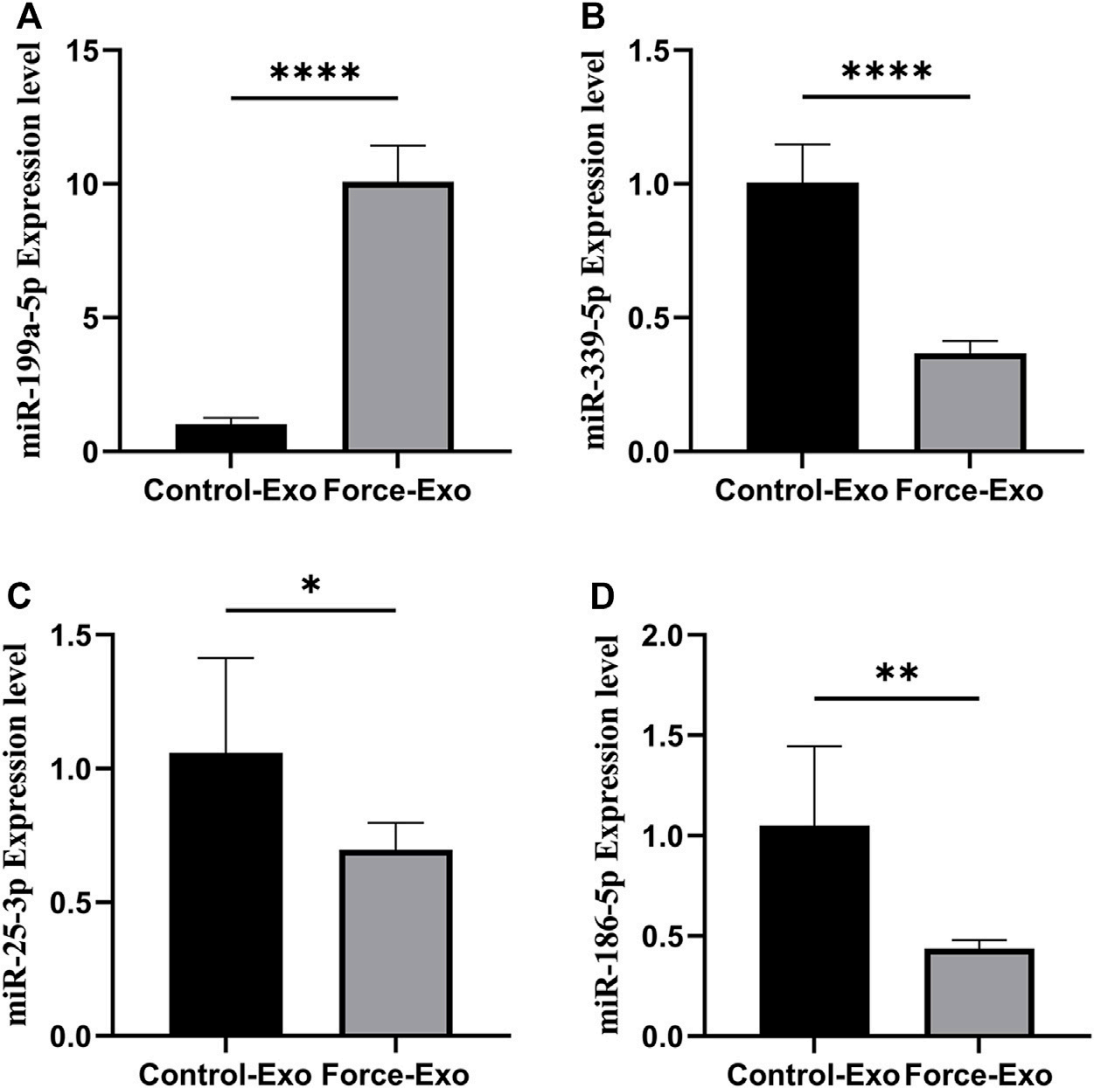
Validation of differential miRNA expression profiles. **(A)** miR-199a-5P expression in condylar chondrocytes was significantly higher in the Force-Exo group than in the Control-Exo group, N = 3 for each group, ****p < 0.0001. **(B)** miR-339-5P expression in condylar chondrocytes was significantly lower in the Force-Exo group than in the Control-Exo group, N = 3 for each group, ****p < 0.0001. **(C)** miR-25-3P expression in condylar chondrocytes was significantly lower in the Force-Exo group than in the Control-Exo group, N = 3 for each group, *p < 0.05. **(D)** miR-186-5P expression in condylar chondrocytes was significantly lower in the Force-Exo group than in the Control-Exo group, N = 3 for each group, **p < 0.01.

Four miRNAs with significant differences in expression between the Force-Exo and Control-Exo groups were selected to verify the miRNA sequencing results. The results of this analysis are shown in Figure 7. The expression level of miR-199a-5p (Figure 7A) in the Force-Exo group was significantly higher than that in the Control-Exo group, and the expression levels of miR-186-5p, miR-339-5p, and miR-25-3p in the Force-Exo group were significantly lower than those in the Control-Exo group (Figures 7B–D). The expression level results of the miRNAs determined by RT-qPCR were consistent with their sequencing results, confirming the reliability and reproducibility of the miRNA sequencing method. The primer sequences used for RT-qPCR are shown in Table 3.

### GO and KEGG analyses

**FIGURE 8:**
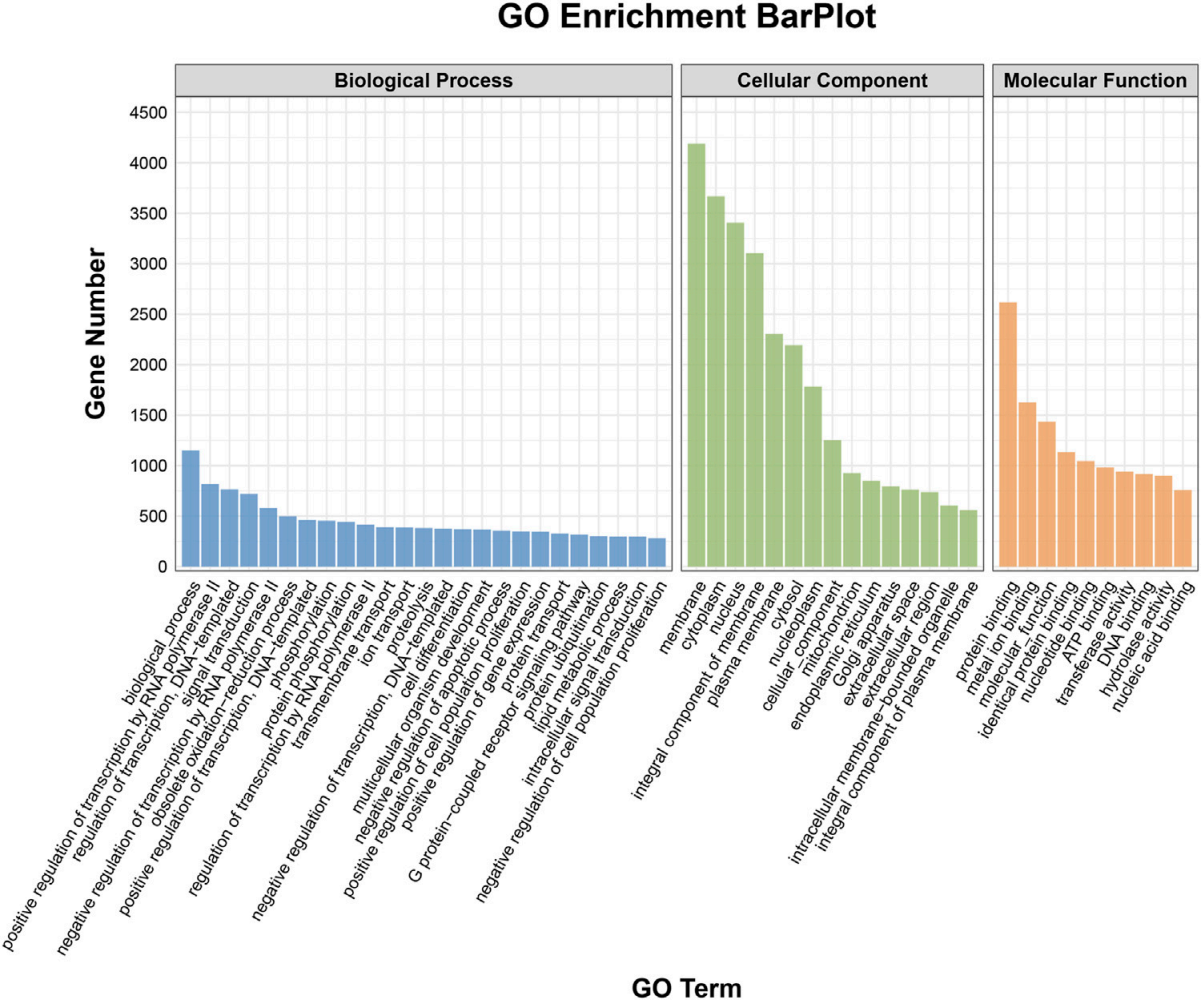
GO annotation enrichment analysis of genes encoding miRNAs expressed differentially between the Force-Exo and Control-Exo groups. Blue represents biological processes, green represents cellular components, and orange represents molecular functions.

**FIGURE 9:**
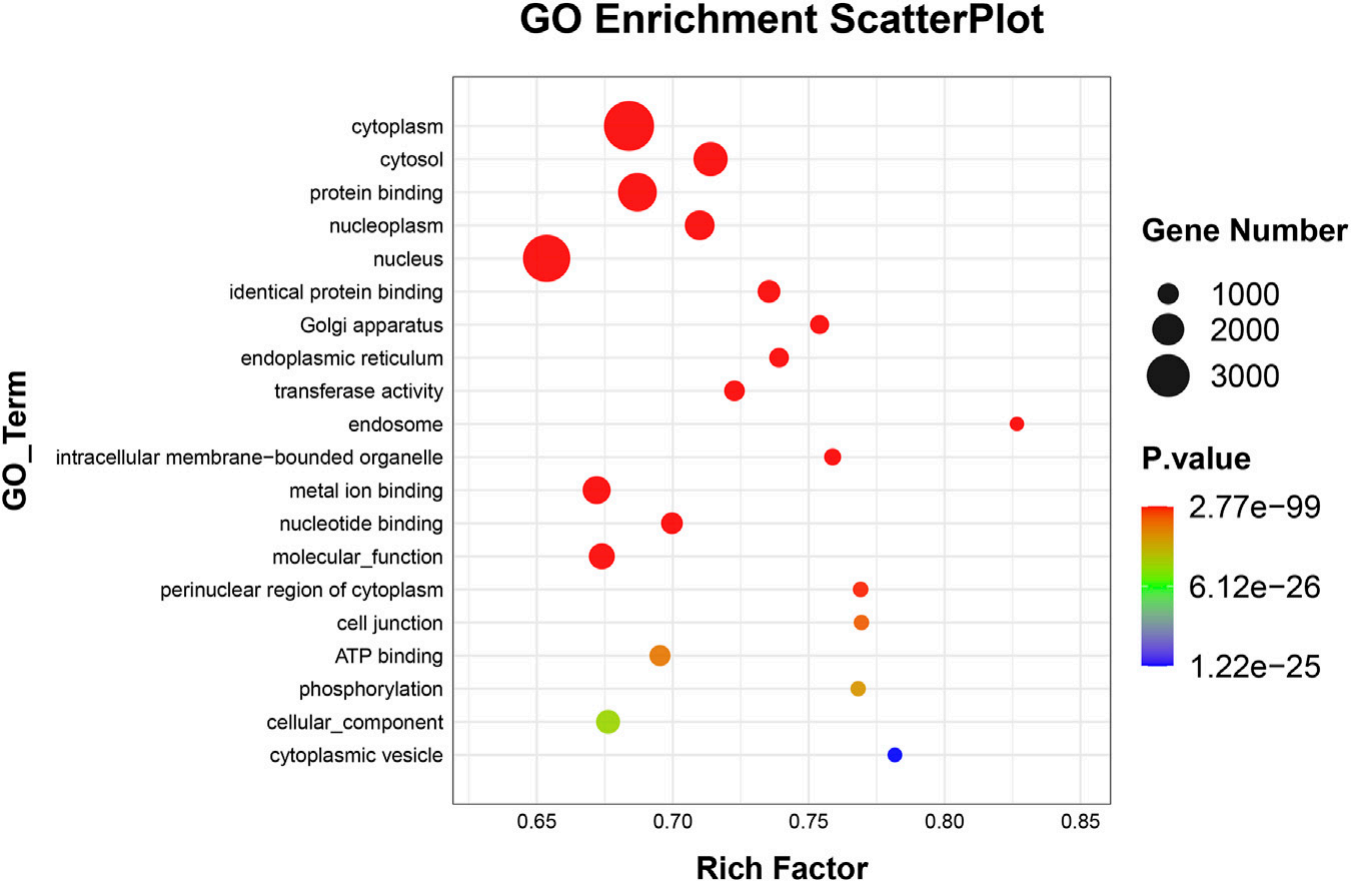
GO enrichment of genes encoding miRNAs expressed differentially between the Force-Exo and Control-Exo groups. Bubble size represents the number of differential genes annotated for a GO term. The redder the color, the smaller the significance and higher the enrichment level.

**FIGURE 10:**
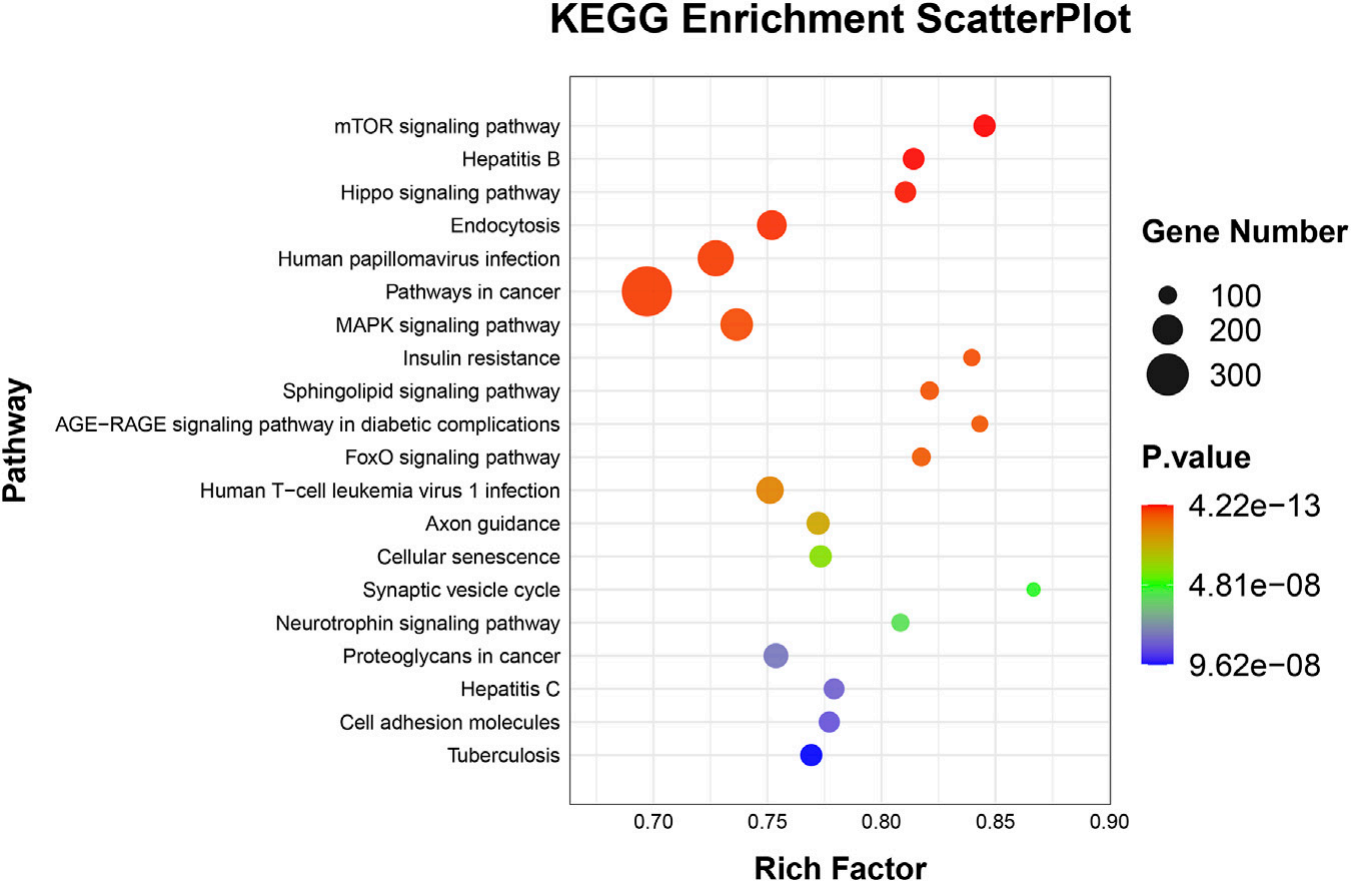
Target genes of miRNAs expressed significantly differentially between the teo MCC-Exo groups were enriched in KEGG pathways. Bubble size represents the number of differential genes annotated for a certain pathway. The redder the color, the smaller the significance and higher the enrichment level.

**FIGURE 11:**
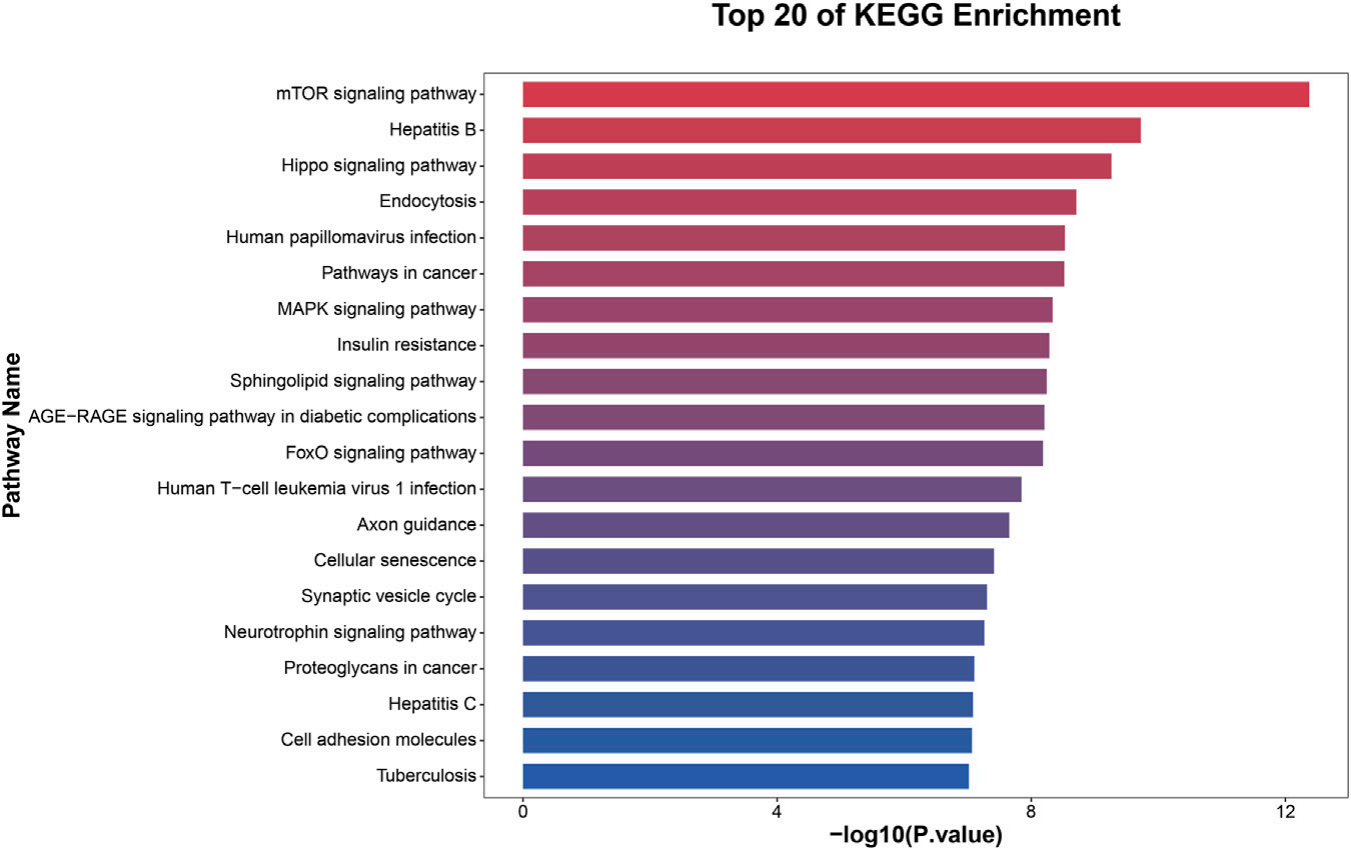
KEGG pathway significance ranking map (*p* ≤ 0.05). The *x*-axis presents enrichment ratios, and the *y*-axis presents KEGG pathways.

To elucidate the activities and interactions of exosomal miRNAs that showed significant differential expression under cellular tensile stress, GO annotation and enrichment and KEGG pathway analysis were performed. The genes encoding miRNAs in the Force-Exo group were assigned GO terms based on sequence homology. There are three types of GO terms: cellular component, biological process, and molecular function. The results of the GO analysis revealed that biological process terms primarily consisted biological process, positive regulation of transcription by RNA polymerase II, transcription regulation, DNA-templated; cellular component terms primarily consisted membrane, cytoplasm, and nucleus; and molecular function terms primarily consisted protein binding, metal ion binding, and molecular function. (Figure 8). There was significant GO enrichment in cellular components, including the cytoplasm, cytosol, and membrane (Figure 9), maybe because of the formation and secretion of exosomes after the fusion of polycystic bodies with the cell surface. Pathway enrichment analysis showed that the genes encoding miRNAs in the Force-Exo group belonged to various pathways. The target genes of differentially expressed miRNAs showed the highest enrichment ratio in the mTOR signaling pathway (Figure 10). Among the top 20 significant KEGG pathways, the mTOR signaling pathway was the most significant, followed by the hepatitis B and Hippo signaling pathways (Figure 11). The abovementioned results can provide a reference for the exploration of osteogenesis-related signaling pathways in the future.

## Discussion

Class II malocclusion in China is highly common in clinical practice. Surveys have shown that the prevalence of Class II malocclusion among children in China varies slightly at each dental stage, ranging from 25.8% at early mixed dentition to 19.4% at early permanent dentition (Minkui Fu et al., 2002). For the orthodontic treatment of patients with Class II malocclusion at Growth and development period (around puberty), early functional orthopedic treatments, such as Activator (Oh et al., 2020), Herbst (Souki et al., 2017; Wei et al., 2020), Frankel II (Galluccio et al., 2021), and Twin block (Uy-Co et al., 2010), are usually used to induce displacement of the mandibular condyle in the downward and forward directions to promote forward mandibular growth, reduce the facial shape of the Class II skeleton, and reduce the need for later surgery. However, there is still a lack of research on the mechanism through which tensile stress promotes the growth and development of condylar cartilage. Recent studies on exosomes have shown that exosomes and their miRNAs play an important role in intercellular signaling and biological activities and that there are abundant exosomes in the condylar cartilage and subchondral bone; it has also been shown that the development of osteoarthritis of the temporomandibular joint is closely related to exosomes in the tissue environment (Zhang et al., 2019). Therefore, in this study, we extracted exosomes from condylar chondrocytes under tensile stress for the first time and co-cultured them with rat condylar chondrocytes to investigate the role played by exosomes in condylar cartilage osteogenesis.

MiRNAs are small non-coding single-stranded RNAs containing about 20–24 nucleotides and encoded by endogenous genes; they are widely found in both plants and animals (Bartel and Chen, 2004; Mendell and Olson, 2012). MiRNAs play an important role in gene regulation. Primary miRNA transcripts in the nucleus are encoded by polymerases I and III and are later sheared by the ribonuclease III Drosha and Dicer to become mature miRNAs that can bind to the RNA-induced silencing complex (RISC) (Li and Ruan, 2009). RISC can cause mRNA degradation or translation inhibition by binding to the 3′-untranslated region (3′-UTR) of target gene mRNAs, thus affecting cell proliferation, differentiation, apoptosis, metabolism, and biological functions (Tuddenham et al., 2006). To date, more than 1,000 miRNAs have been identified in humans, and it is estimated that these target approximately 60% of total human mRNAs (Kopańska et al., 2017). A single miRNA can regulate multiple target genes, and a single target gene can be regulated by multiple miRNAs (Iorio and Croce, 2012).

Exosomes are cystic vesicles composed of lipid bilayers that can be secreted by a variety of cells and are widely present in body fluids, such as blood, saliva, lymphatic fluid, and cerebrospinal fluid (Raposo and Stoorvogel, 2013; Brinton et al., 2015; Keerthikumar et al., 2016). Exosomes play a range of biological roles, including intercellular communications and infectious material distribution, and can also serve as carriers for miRNA delivery to target cells. Exosomes and their recipient cells interact *via* three mechanisms: first, direct interaction between exosome transmembrane proteins and target cell signaling receptors; second, fusion of exosomes with the plasma membranes of recipient cells for content delivery to the cytoplasm; third, internalization of exosomes into recipient resulting in two fates—fusion of phagocytosed exosomes with endonucleosomes to undergo cytolysis, allowing exosome contents to enter the cytoplasm and fusion of the endosomes of the phagocytosed exosomes to form lysosomes that degrade to release exosomal contents into the recipient cell cytoplasm (Munich et al., 2012; Tian et al., 2013). Exosomes, as a functional component among cell-secreted vesicles, have high scientific and clinical applicability because they avoid the problem of unstable effects generated by the direct use of cells (Ai et al., 2022). Studies have demonstrated that mechanical stimulation can alter the composition of miRNAs in exosomes; for example, choroidal trophoblast cells under different types of oxygen tension show significant changes in the expression of miRNAs related to cell migration (Truong et al., 2017), and the expression of miRNAs in exosomes secreted by periodontal ligament stem cells change significantly under cyclic tension to regulate macrophages through the IL-1β/NF-κB signaling pathway and affect the maintenance of periodontal homeostasis (Wang et al., 2019). These findings suggest the existence of a stress signaling-sensitive mechanism for the assembly of cellular exosomal inclusions, which involves the regulation of certain exosomal miRNAs (Akerman et al., 2019). In this study, we co-cultured exosomes cultured under normal and tensile stress conditions with rat condylar chondrocytes and found that the results of differential miRNA expression between the two conditions after co-culture were consistent with the sequencing results.

Condylar cartilage osteogenesis is mainly intrachondral osteogenesis. During this process, MSCs aggregate and differentiate into chondrocytes, which proliferate and secrete a cartilage matrix rich in type II collagen and proteoglycans. In response to a signal stimulus, the proliferating chondrocytes stop proliferating and hypertrophy, secreting a matrix rich in type X collagen; this is followed by vascularization, indicating the onset of osteogenesis in cartilage (Kronenberg, 2003). The precise regulation of the above process depends on the sequential expression of a series of factors, among which *sox9* plays a complex regulatory role through different signal transduction pathways (Dy et al., 2012). *Sox9* can promote the differentiation of mesenchymal cells into chondrocytes and chondrocyte proliferation. It can also delay the pre-hypertrophy of chondrocytes by down-regulating the expression of *Runx2* and *Mef2c* and can induce the hypertrophy of cartilage cells by other means (Dy et al., 2012). *Runx2* is required for condylar chondrocyte proliferation and hypertrophy and postnatal condylar cartilage growth and homeostasis. Studies have shown that Runx2 is maintained at high levels of expression during condylar growth and development (Huang et al., 2020). *Runx2* tightly regulates endochondral bone formation, mainly through a feedback loop involving key factors for chondrocyte differentiation, such as IHH and PTHrP (Li et al., 2015; Kim et al., 2020), but it also influences endochondral vascular invasion and cartilage differentiation into bone by regulating the expression of cytokines, such as MMP13 and VEFG-A in prehypertrophic and hypertrophic chondrocytes (Wang et al., 2004). Runx2 is also involved in Notch signaling (Xiao et al., 2019) and the Wnt/β catenin signaling pathway (Dong et al., 2006), affecting chondrocyte proliferation and differentiation. It plays an important role in regulating chondrocyte-derived subchondral bone remodeling (Chen et al., 2014). Therefore, we selected *Runx2* and *Sox9* as indicators of chondrocyte osteogenic differentiation in this study. Recently, it was found that exosomes secreted by chondrocytes are involved in pathological mineralization and intercellular communication in OA cartilage, affecting cartilage maintenance and the development of osteoarthritis (Ni et al., 2020). These exosomes have a dual role in OA; exosomes from healthy chondrocytes are highly bioactive and can delay the progression of osteoarthritis by regulating the penetration of M2 macrophages to reverse mitochondrial dysfunction and restore the immune response (Zheng et al., 2019). They contain miR-8485, which inhibits the expression of glycogen synthase-3β, activates the Wnt/β-catenin pathway, and promotes the differentiation of bone marrow mesenchymal stem cells to form cartilage (Li et al., 2020). Conversely, exosomes from osteoarthritic chondrocytes cause chondrocyte apoptosis, inhibit cell proliferation, stimulate activation of inflammatory vesicles, and increase mature interleukin 1β in macrophages by promoting miR-449A-5p/ATG4B-mediated autophagy (Ni et al., 2019). In this study, Force-Exo and Control-Exo group exosomes were co-cultured with rat condylar chondrocytes; Force-Exo exosomes were found to promote the expression of *Runx2* and *Sox9* in condylar chondrocytes, indicating that tensile stress stimulation can alter the expression of certain osteogenesis-related miRNAs in MCC-Exos and can promote the osteogenic differentiation of condylar cartilage. This provides a new idea for exosome modification for the treatment of OA.

In this study, we found that miR-199-5p expression was significantly increased in exosomes from cells cultured under tensile stress and may target the expression of *ALP*, *BMP3*, and *ECE1* to affect osteogenesis-related functions. It was found that miR-199a-5p is expressed in both osteoblasts and chondrocytes (Laine et al., 2012) and is closely associated with bone formation. When bone density is reduced, a similar reduction in serum miR-199a-5p expression can be detected (Yi, 2017). miR-199a-5p targets Ten-Eleven Translocation 2 (*TET2*) and enhances alkaline phosphatase (*ALP*) activity as well as promotes the upregulation of osteogenic factors, such as Runx2 and OCN, by negatively regulating *TET2* thereby inducing osteogenic differentiation of hBSMCs (Laine et al., 2012; Qi et al., 2020). It can also target *ECE1* and promote MC3T3-E1 cell proliferation, osteogenic differentiation, and mineralization by downregulating *ECE1* expression, which can promote bone injury repair (Liu Chong et al., 2020). miR-199a-5p can also promote osteogenesis by inhibiting the HIF1α-Twist pathway (Chen et al., 2015). Also, miR-199a-5p plays an important role in osteoarthritis. It was found that miR-199a-5p also inhibits visfatin (a pro-inflammatory adipokine)-induced IL-6 and TNF-α production to mitigate the progression of osteoarthritis (Wu et al., 2018). Similarly, its sister miRNA miR-199a-3p targets cyclooxygenase-2 (*Cox-2*) to stabilize cartilage morphology by inhibiting interleukin-1β (IL-1β)-induced *COX-2* expression in osteoarthritis chondrocytes (Akhtar and Haqqi, 2012). In summary, miR-199a-5p is closely associated with osteogenesis. In this study, tension stress could effectively increase the expression of MCC-Exo miR-199a-5p, laying the foundation for its in-depth study in cartilage osteogenesis-related fields.

The significantly down-regulated miRNAs in the Forec-Exo group in this study, such as miR-186-5p, miR-339-5p, and miR-25-3p, also play an important role in osteogenesis. It was found that miR-186-5p inhibits phosphatidylinositol three kinase (Pi3K)/protein kinase B (AKT) signaling by targeting cXcl13, downregulates osteoblast-specific markers, and reduces the viability of human bone marrow mesenchymal stem cells, thereby affecting bone regeneration and repair (Xu et al., 2019). circRNA_0001795 overexpression promotes hBMSC osteogenic differentiation, whereas miR-339-5p can inhibit the expression of circRNA_0001795 by downregulating yes-associated protein 1 (YAP1), thereby reducing *ALP* activity and inhibiting osteogenic differentiation of hBMSCs (Li et al., 2022). It has been reported that miR-339-5p is highly expressed in osteoporotic patients and suppresses the expression of osteogenic genes, such as *Runx2*, *OCN*, and *OPN* (Zhong Yan et al., 2021). Although existing studies have not identified the effect of miR-25-3p on osteogenesis, a miRWalk search showed that miR-25-3p may inhibit osteogenesis by downregulating genes involved in osteoblast differentiation (*FOSL1*, *RUNX2*, *WNT9A*, *BMP2K*) (Legrand et al., 2020).

KEGG pathway enrichment analysis showed that the target genes of miRNAs differentially expressed between the two MCC-Exo groups in this study were enriched in multiple pathways under control and tensile stress conditions; this finding has certain research prospects. The most enriched pathway was the mTOR signaling pathway. The mammalian target of rapamycin (mTOR) is a serine/threonine kinase. Recent studies have found that mTOR/Rictor/AKT and mTOR/raptor regulate osteoclast differentiation by promoting osteoclast fusion and increasing osteoclast cell size (Tiedemann et al., 2017). OPG/RANK/RANKL, Ephrin2/ephB4, RANKL/LGR4/RANK, Fas/FasL, and other signaling pathways are also involved in bone remodeling (Chen et al., 2018), and most of them are related to the mTOR signaling pathway (Murugan, 2019). Li (Xiang et al., 2020) et al. found that miR-142-5p, as a CXCR4-targeted miRNA, could alleviate SDF-1-induced chondrocyte apoptosis and cartilage degradation by inactivating the MAPK signaling pathway. Dong (Dong et al., 2020; Pan et al., 2021) also confirmed that the MAPK signaling pathway is involved in cartilage degradation and repair.

Although this study successfully demonstrated that Force-Exo promotes the osteogenic differentiation of condylar chondrocytes, the cellular stress model used in this study does not fully simulate the changes in the surrounding stress environment during the growth and development or treatment of the human condyle. The stress environment around the condyle is intricate and continuously changing. In the future, cellular mechanics experiments with cyclic reciprocal *in vitro* tensile stress stimulation and compressive stress stimulation should be carried out. Meanwhile, this study is a cellular-level mechanics study and an exosomal genetics-level study and can thus be useful for clinical purposes. However, further animal studies and corresponding clinical trial studies are still needed to more precisely investigate the effects of stress on condylar growth and development and the mechanism of functional orthosis-guided mandibular growth and remodeling.

## Conclusion

This study further analyzed and refined the understanding of the molecular mechanism through which tensile stress promotes condylar growth and development from a new perspective. It demonstrated that exosomes play an important role in information exchange between condylar chondrocytes, screened miRNAs that are significantly differentially expressed in condylar chondrocyte exosomes under tensile stress, and predicted the osteogenesis promoting effect of these miRNAs by bioinformatics analysis, providing a reference and theoretical basis for further mechanobiological study of condylar chondrocytes. However, more in-depth *in vivo* studies and corresponding clinical trial studies are needed to investigate more precisely, the effect of stress on condylar growth and development and the mechanism of functional orthosis-guided mandibular growth and remodeling.

## Data Availability

The original contributions presented in the study are included in the article/Supplementary Material, further inquiries can be directed to the corresponding author.
