# Supplementary figures and images for: Effect of condylar chondrocyte exosomes on condylar cartilage osteogenesis in rats under tensile stress
Source: Front Bioeng Biotechnol. 2022 Dec 6;10:1061855. doi: 10.3389/fbioe.2022.1061855 (PMC9766957; doi:10.3389/fbioe.2022.1061855)

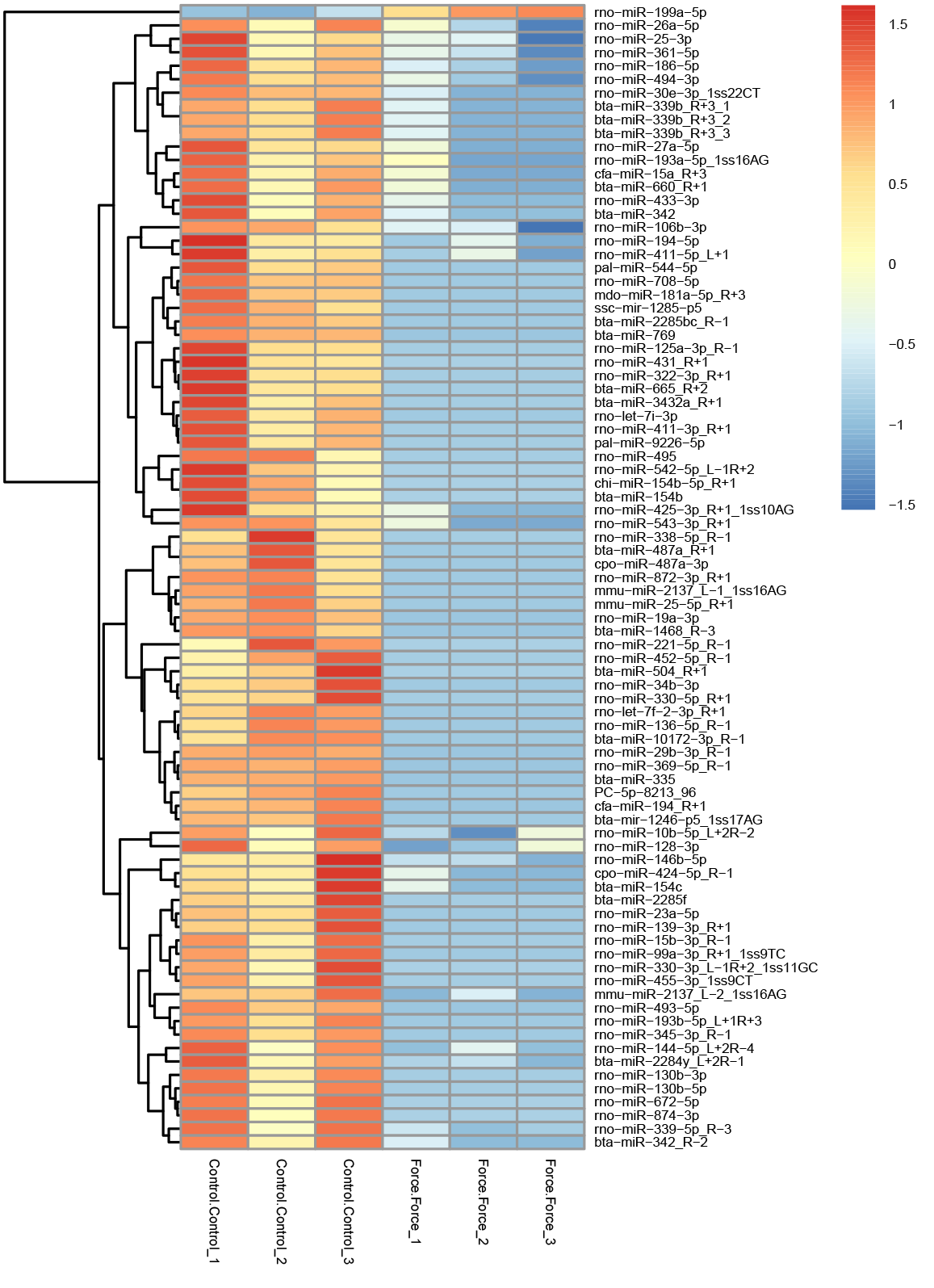

Supplement: Supplementary file 1 [file DataSheet1.ZIP › 压缩图片/figure4.tif]

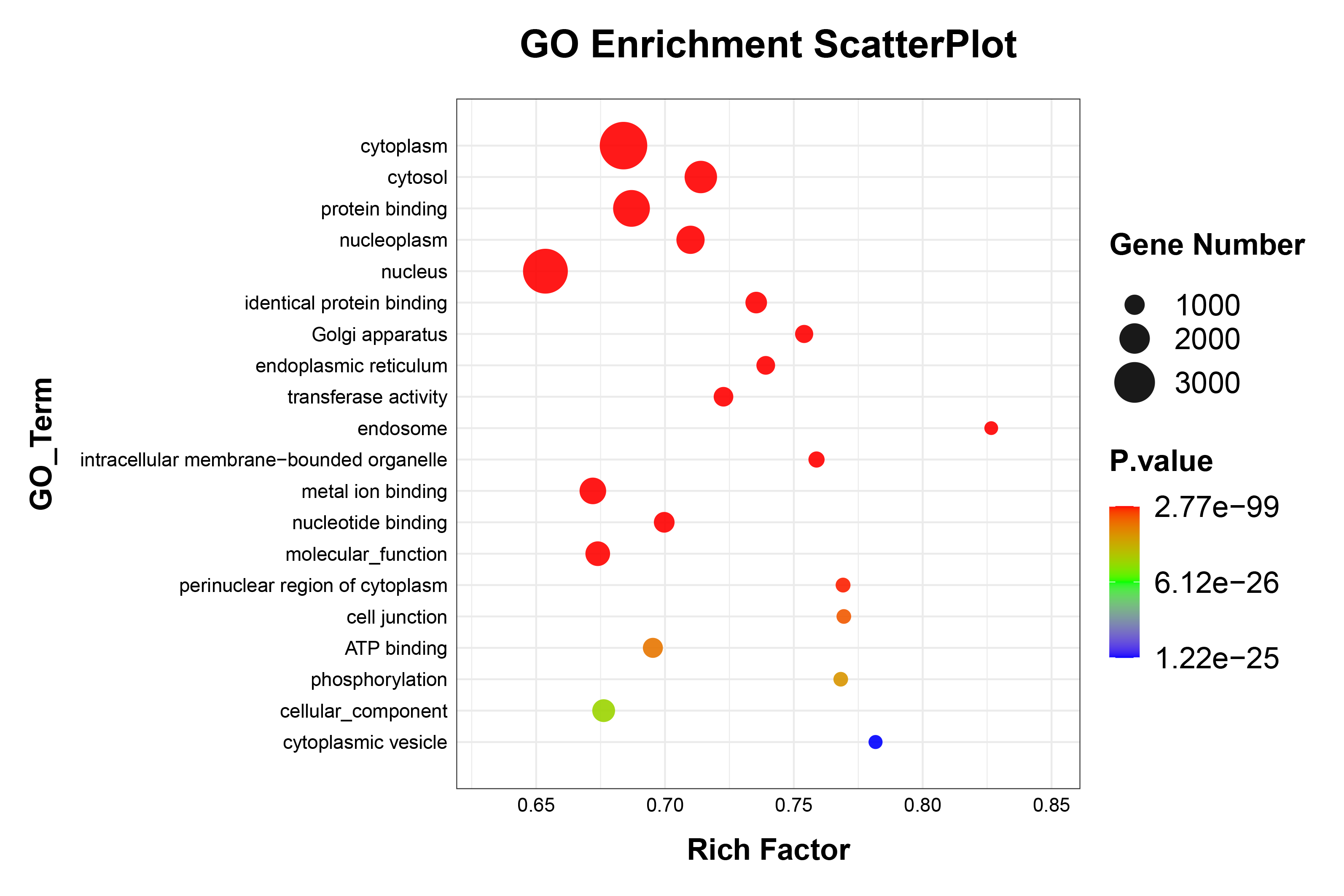

Supplement: Supplementary file 1 [file DataSheet1.ZIP › 压缩图片/figure9.tif]

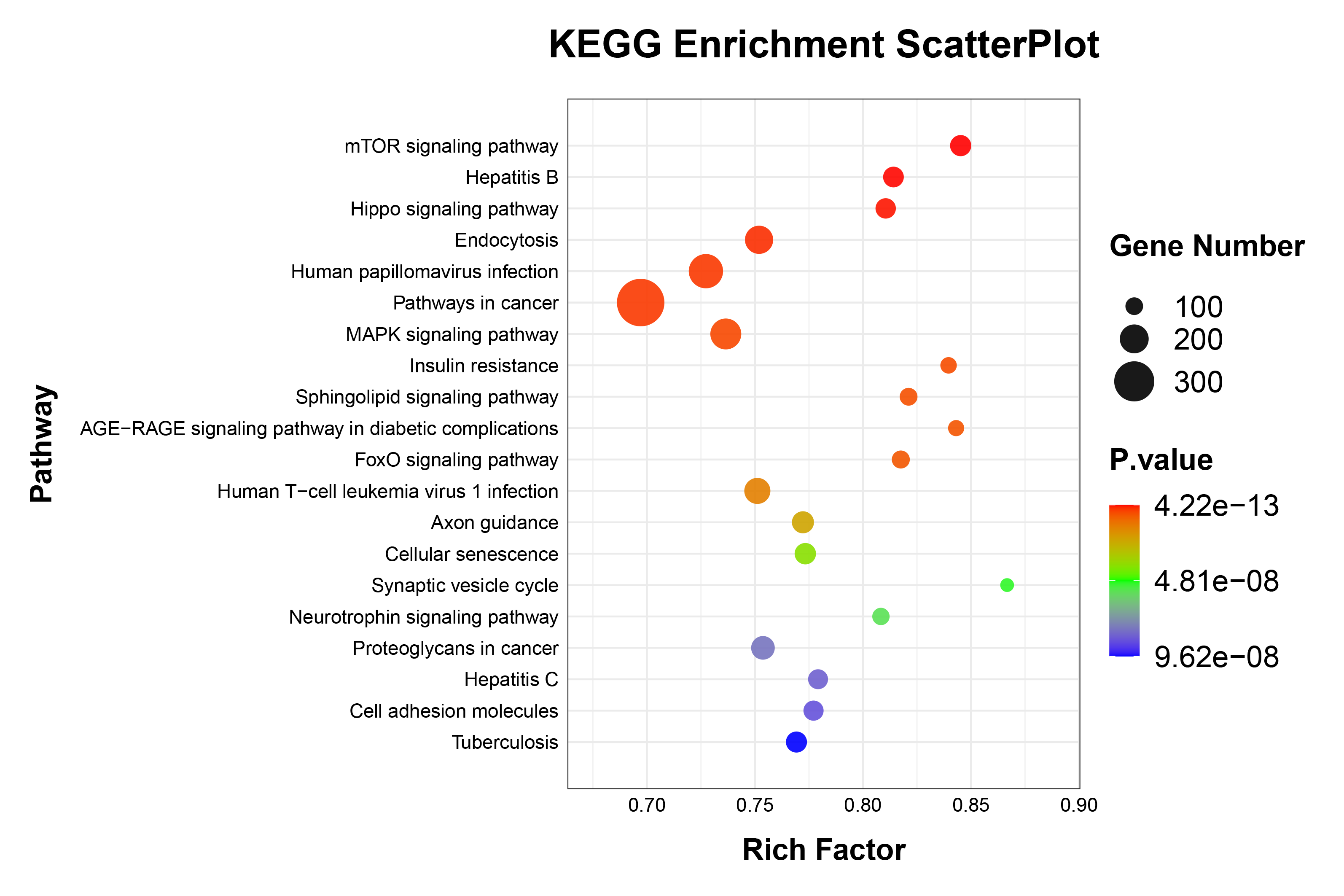

Supplement: Supplementary file 1 [file DataSheet1.ZIP › 压缩图片/figure10.tif]

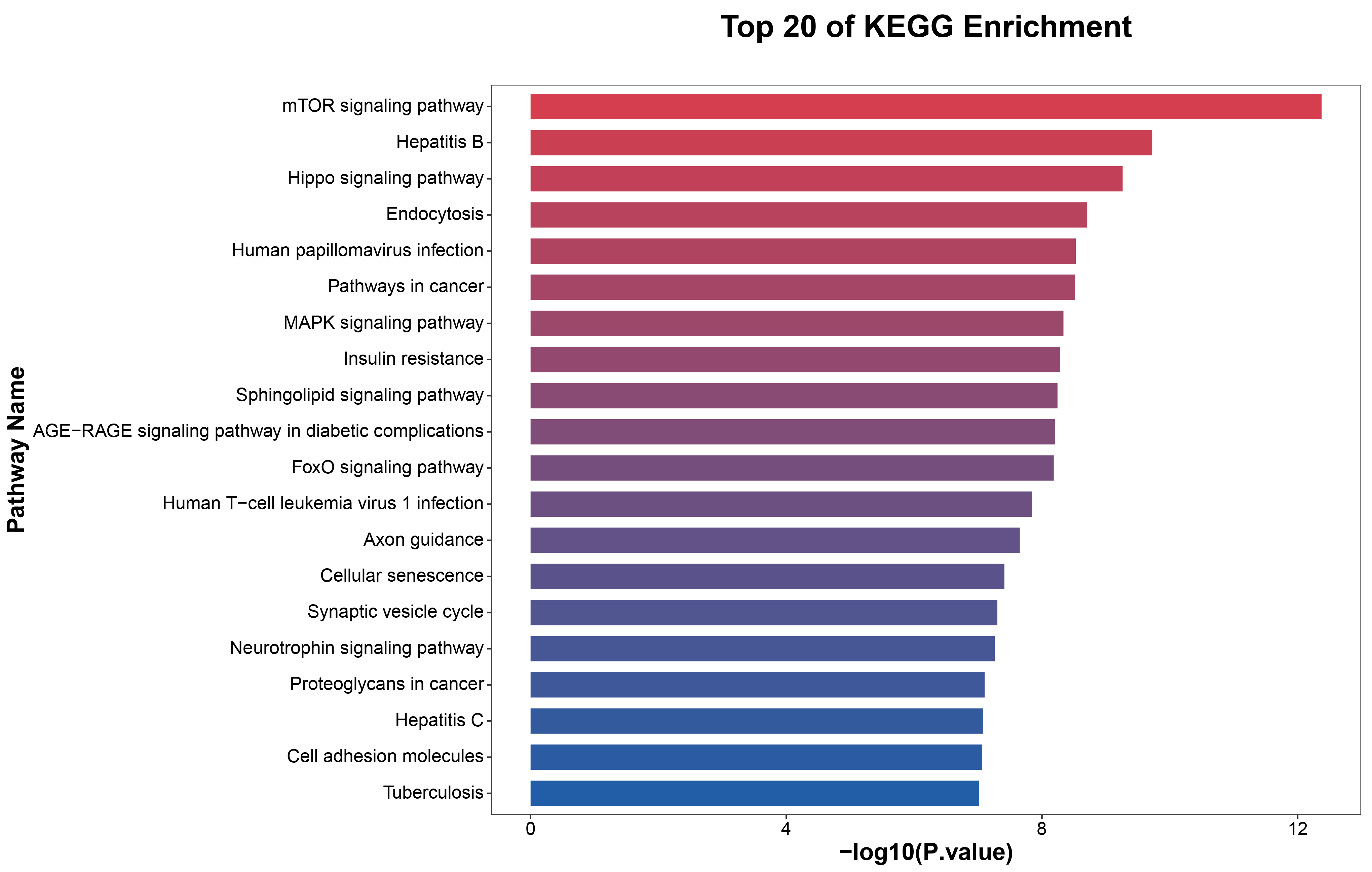

Supplement: Supplementary file 1 [file DataSheet1.ZIP › 压缩图片/figure11.tif]

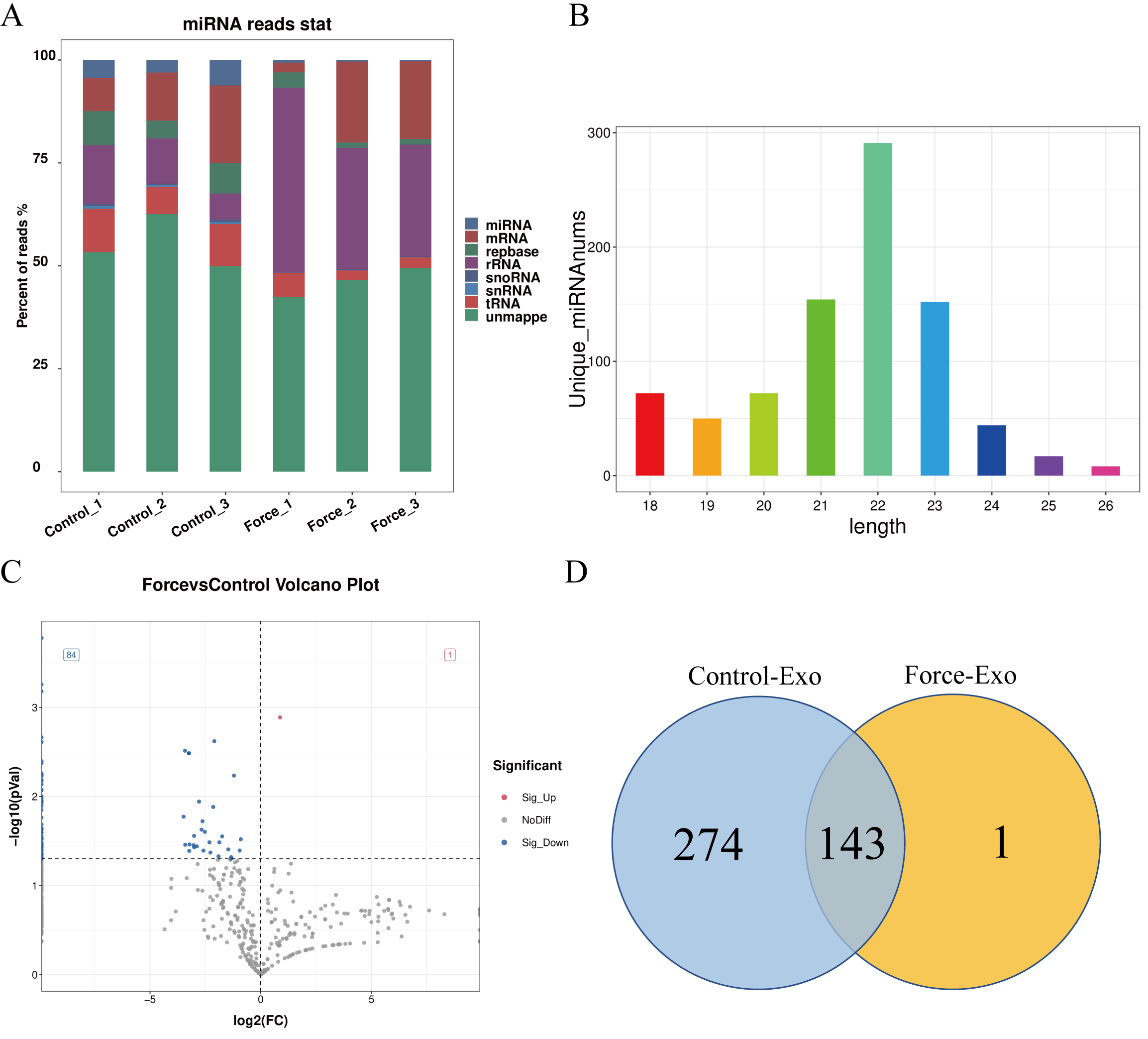

Supplement: Supplementary file 1 [file DataSheet1.ZIP › 压缩图片/R-figure 3.tif]

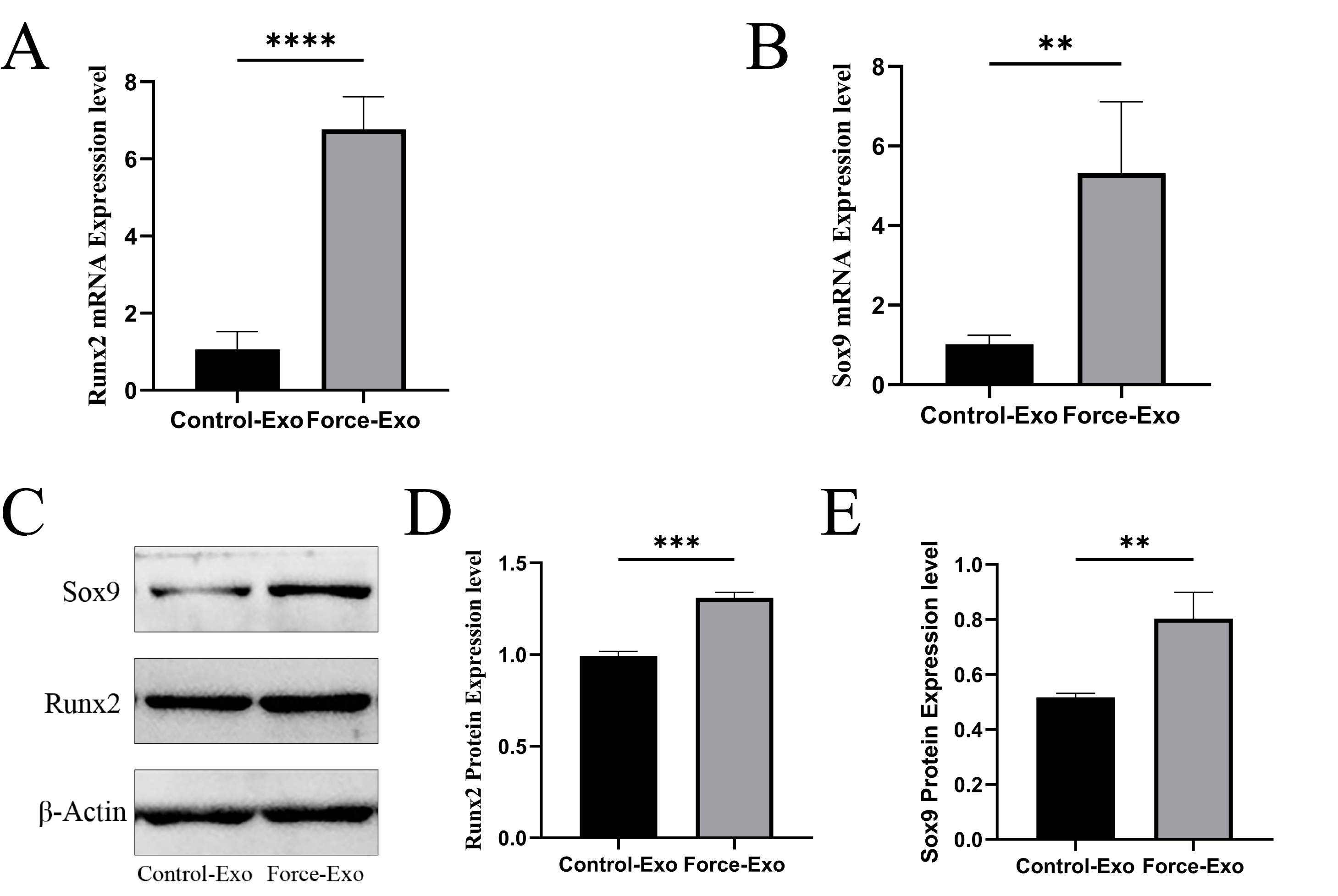

Supplement: Supplementary file 1 [file DataSheet1.ZIP › 压缩图片/R-figure6.tif]

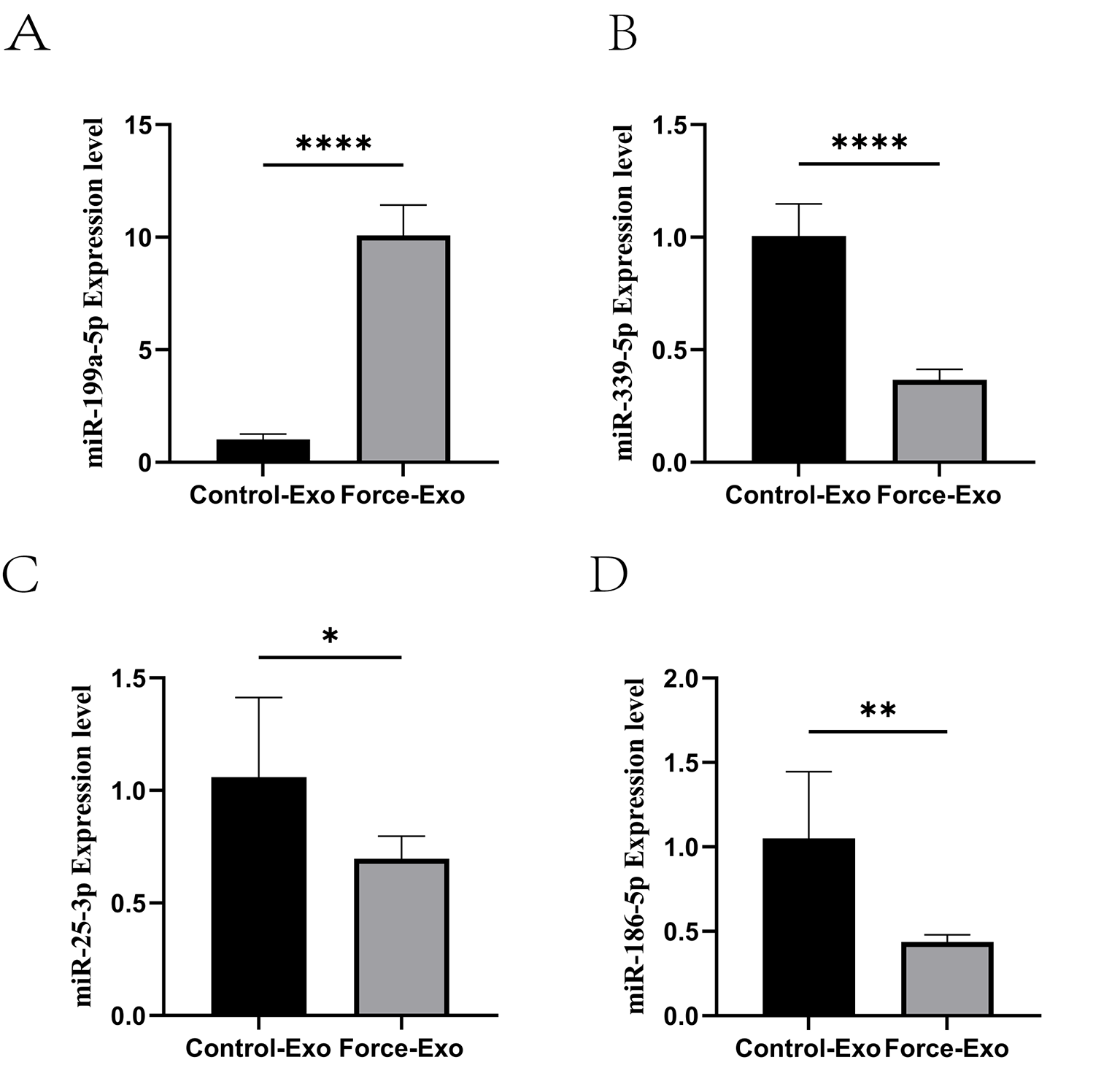

Supplement: Supplementary file 1 [file DataSheet1.ZIP › 压缩图片/R-figure7.tif]

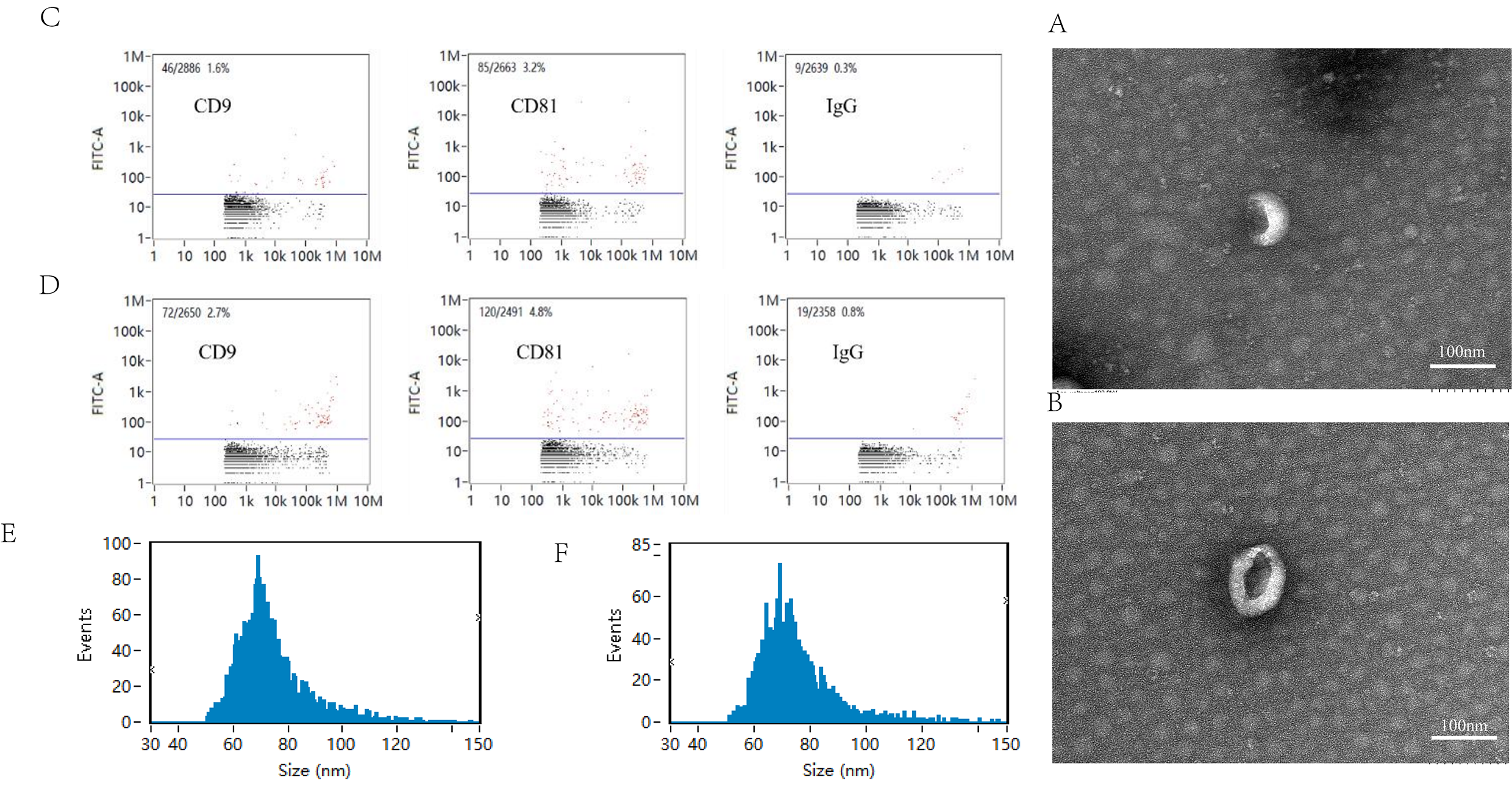

Supplement: Supplementary file 1 [file DataSheet1.ZIP › 压缩图片/R-figure 2.tif]

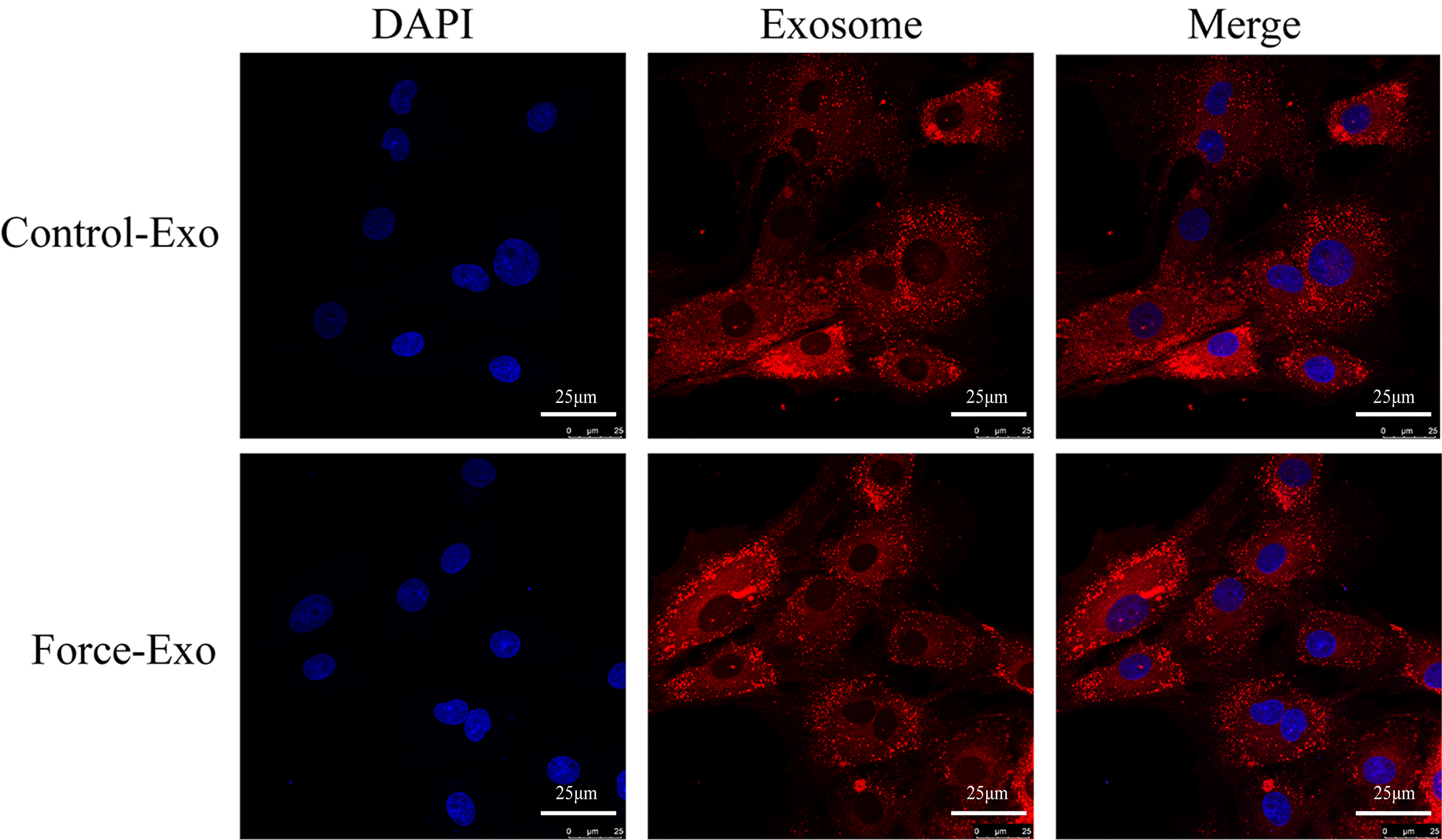

Supplement: Supplementary file 1 [file DataSheet1.ZIP › 压缩图片/R-figure5.tif]

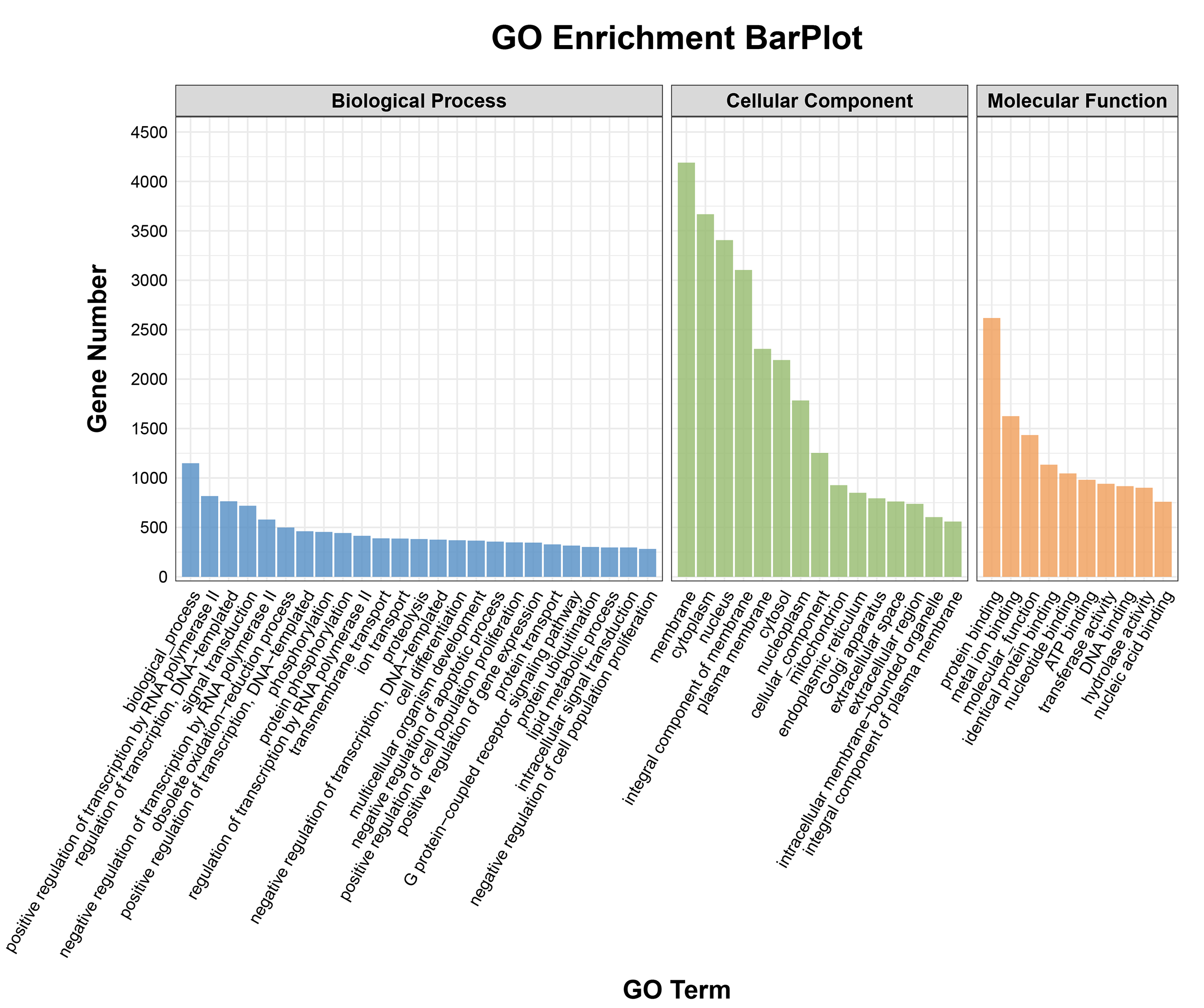

Supplement: Supplementary file 1 [file DataSheet1.ZIP › 压缩图片/figure8.tif]

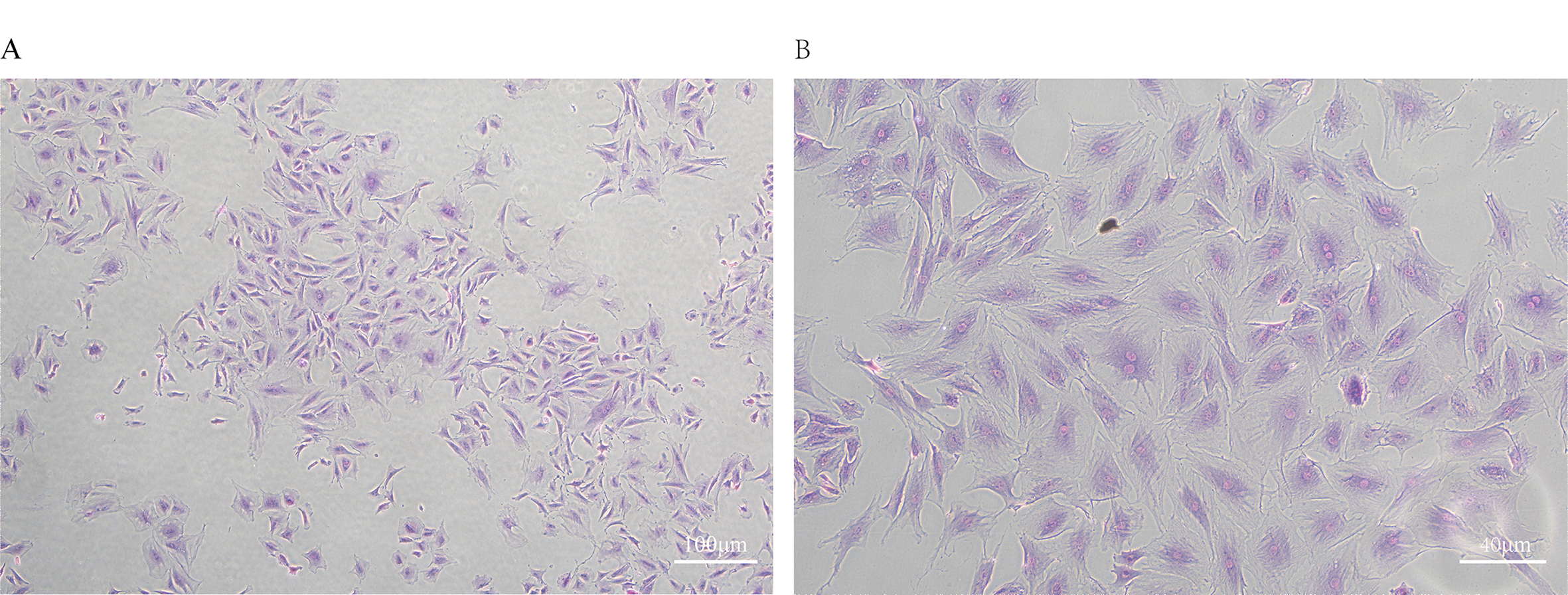

Supplement: Supplementary file 1 [file DataSheet1.ZIP › 压缩图片/R-figure1.tif]
